# A Lipid-Structured Model of Atherosclerotic Plaque Macrophages with Lipid-Dependent Kinetics

**DOI:** 10.1007/s11538-023-01193-w

**Published:** 2023-08-15

**Authors:** Michael G. Watson, Keith L. Chambers, Mary R. Myerscough

**Affiliations:** 1 School of Mathematics and Statistics, University of New South Wales, Kensington, NSW 2052 Australia; 2 Wolfson Centre for Mathematical Biology, Mathematical Institute, University of Oxford, Oxford, Oxfordshire OX2 6GG UK; 3 School of Mathematics and Statistics, University of Sydney, Camperdown, NSW 2006 Australia

**Keywords:** Atherosclerosis, Macrophage, Lipid, Partial integro-differential equation, Structured population model

## Abstract

Atherosclerotic plaques are fatty growths in artery walls that cause heart attacks and strokes. Plaque formation is driven by macrophages that are recruited to the artery wall. These cells consume and remove blood-derived lipids, such as modified low-density lipoprotein. Ineffective lipid removal, due to macrophage death and other factors, leads to the accumulation of lipid-loaded macrophages and formation of a necrotic lipid core. Experimental observations suggest that macrophage functionality varies with the extent of lipid loading. However, little is known about the influence of macrophage lipid loads on plaque fate. Extending work by Ford et al. (J Theor Biol 479:48–63, 2019) and Chambers et al. (A lipid-structured model of atherosclerosis with macrophage proliferation, 2022), we develop a plaque model where macrophages are structured by their ingested lipid load and behave in a lipid-dependent manner. The model considers several macrophage behaviours, including recruitment to and emigration from the artery wall; proliferation and apotosis; ingestion of plaque lipids; and secondary necrosis of apoptotic cells. We consider apoptosis, emigration and proliferation to be lipid-dependent and we model these effects using experimentally informed functions of the internalised lipid load. Our results demonstrate that lipid-dependent macrophage behaviour can substantially alter plaque fate by changing both the total quantity of lipid in the plaque and the distribution of lipid between the live cells, dead cells and necrotic core. The consequences of macrophage lipid-dependence are often unpredictable because lipid-dependent effects introduce subtle, nonlinear interactions between the modelled cell behaviours. These observations highlight the importance of mathematical modelling in unravelling the complexities of macrophage lipid accumulation during atherosclerotic plaque formation.

## Introduction

Atherosclerotic plaques are localised accumulations of cells, lipids and associated debris that form in the lining of major arteries (Hansson and Libby 2006). Plaques are initiated when blood-borne low-density lipoprotein (LDL) penetrates the endothelium of the artery and is deposited in the artery wall (Tabas et al. 2007). Once inside the artery wall, LDL is oxidised or modified in different ways. Accumulation of modified LDL elicits an immune response that attracts circulating monocytes. These monocytes rapidly differentiate into macrophages, which ingest (phagocytose) the modified LDL and stimulate further macrophage recruitment through inflammatory signalling (Moore et al. 2013; Tall and Yvan-Charvet 2015). The death of lipid-loaded macrophages (known as foam cells) creates fatty deposits that may accumulate over time to form a large necrotic core (Lusis 2000). Rupture of a vulnerable plaque can release this necrotic material into the bloodstream and trigger a clotting cascade that causes stroke or myocardial infarction (Lusis 2000; Hansson and Libby 2006).

Not all plaques progress to become clinically dangerous. Many simply resolve naturally or evolve towards a benign, non-resolving state (Bäck et al. 2019). The fate of a plaque is largely determined by the interaction between macrophages and lipids in the artery wall and, in particular, the relative rates at which these constituents enter and leave the tissue (Moore et al. 2013). In addition to recruitment by inflammatory signalling, the plaque macrophage population can be increased by local proliferation (Robbins et al. 2013; Lhoták et al. 2016). On the other hand, plaque macrophage numbers can be reduced by death (apoptosis) or by emigration out of the wall (Tabas 2010; Llodrá et al. 2004). Since infiltrating LDL becomes bound to the artery wall extracellular matrix (Tabas et al. 2007), the removal of this lipid from the system requires the intervention of macrophages. Lipid internalised by macrophages can be ferried out of the plaque during macrophage emigration or by macrophage offloading to high-density lipoprotein (HDL) in a process known as reverse cholesterol transport (Yvan-Charvet et al. 2010). A further important mechanism, which recycles the cell and lipid content of the plaque, is macrophage efferocytosis (Yin and Heit 2021). As observed *in vitro*, a macrophage can phagocytose an entire apoptotic cell and acquire the dying cell’s ingested lipid (Ford et al. 2019b). Apoptotic cells that are not efficiently cleared by efferocytosis are a source of necrotic material (Kojima et al. 2017).

Experimental results indicate that plaque macrophage behaviour can change with the extent of lipid loading (Tabas and Bornfeldt 2016). Lipid accumulation in plaque macrophages can upregulate the production of the pro-inflammatory signals that are required for monocyte recruitment (Tall and Yvan-Charvet 2015). The cytotoxic effects of excessive lipid ingestion can lead to macrophage apoptosis (Tabas 2002; Feng et al. 2003). Using a detailed transcriptomic study of murine plaque macrophages, Kim et al. (2018) showed that macrophage proliferation is likely to decrease with increasing lipid load. Results from both *in vitro* and *in vivo* studies further suggest that macrophages with larger lipid loads are less likely to emigrate from plaques (Chen et al. 2019; van Gils et al. 2012; Wanschel et al. 2013). This is due to either reduced migration capacity (Chen et al. 2019) or through increased expression of so-called retention factors (van Gils et al. 2012; Wanschel et al. 2013). Defective macrophage efferocytosis, an often cited mechanism of atherosclerotic plaque progression (Thorp and Tabas 2009; Linton et al. 2016), may also arise through lipid-dependent effects; lipid loading can reduce the efficiency of efferocytosis by disrupting relevant signalling pathways (Yin et al. 2020) and excessive lipid acquisition through efferocytosis can lead to cytotoxic macrophage death (Yin and Heit 2021).

Experimental plaque formation data is frequently collected *ex vivo* (e.g., from studies that use the genetically modified, atherosclerosis prone ApoE$^{-/-}$ mouse). Thus, although the lipid-dependent effects noted above have been identified, little is known about how they contribute either individually or collectively to plaque formation dynamics. In this paper, we address this issue by developing a mathematical model to study the impact of lipid-dependent macrophage behaviour on the dynamics and fate of atherosclerotic plaque progression. As will be seen, our findings indicate that lipid-dependent effects can substantially alter the fate of a plaque, often in unpredictable ways. This provides valuable new insight, and emphasises the need to consider lipid-dependent plaque cell behaviour both in future mathematical models of plaque formation and in the interpretation of experimental plaque formation data.

Interest in mathematical modelling of atherosclerotic plaque formation has grown in recent years (Parton et al. 2016; Avgerinos and Neofytou 2019; McAuley 2021). Most work to date has focussed on modelling the inflammatory response of macrophages in the early plaque. Published approaches include spatially-averaged ODE models (Bulelzai and Dubbeldam 2012; Cohen et al. 2014; Islam and Johnston 2016; Thon et al. 2018; Lui and Myerscough 2021), spatially-resolved PDE models (El Khatib et al. 2007; Calvez et al. 2009; Little et al. 2009; Yang et al. 2016; Chalmers et al. 2017; Thon et al. 2019; Silva et al. 2020) and agent-based models (Bhui and Hayenga 2017). It is common to assume that the modelled macrophages have two sub-populations: those with little or no internalised lipid (usually termed macrophages) and those with lots of internalised lipid (usually termed foam cells) (Calvez et al. 2009; Bulelzai and Dubbeldam 2012; Cilla et al. 2014; Hao and Friedman 2014; Islam and Johnston 2016; Yang et al. 2016; Chalmers et al. 2017; Silva et al. 2020). Lipid-dependent macrophage behaviour can be implicitly incorporated in this model framework by assuming that these sub-populations have, for example, different rates of lipid consumption (Calvez et al. 2009; Bulelzai and Dubbeldam 2012; Cilla et al. 2014; Hao and Friedman 2014; Islam and Johnston 2016; Silva et al. 2020), migration (Calvez et al. 2009; Cilla et al. 2014; Yang et al. 2016; Chalmers et al. 2017; Silva et al. 2020) or apoptosis (Hao and Friedman 2014; Islam and Johnston 2016; Silva et al. 2020). An alternative approach is to model macrophages as a single population and track the population’s total ingested lipid content (Little et al. 2009; Cohen et al. 2014; Thon et al. 2018, 2019; Lui and Myerscough 2021). Here, lipid-dependent effects can be included at the population-level by assuming that macrophage behaviour depends on the average ingested lipid load (Thon et al. 2018, 2019). A more natural means to model lipid accumulation in plaque macrophage populations, including with lipid-dependent effects, is to use a population model in which macrophages are physiologically structured by their internalised lipid content. Lipid-structured models of plaque macrophages have been developed by Ford et al. (2019a), Chambers et al. (2022), and Meunier and Muller (2019).

The model by Ford et al. (2019a) uses a system of partial integro-differential equations to study how internalised lipid loads are distributed in live and apoptotic plaque macrophage populations, and how this influences necrotic core formation. Possible behaviours of live macrophages in the model include: (i) apoptosis; (ii) emigration from the plaque; (iii) LDL and necrotic lipid phagocytosis; (iv) lipid offloading to HDL; and (v) efferocytosis of apoptotic cells. Simulations and analysis of this model demonstrate the important roles of emigration and efferocytosis in the prevention of necrotic core growth. Efferocytosis is identified as a double-edged sword, however, as it can drive ingested lipid loads to become excessively large [as confirmed experimentally in Ford et al. (2019b)]. Chambers et al. (2022) extended the Ford model to include macrophage proliferation. This introduces an additional means of reducing cell lipid loads as internalised lipid in the parent cell is split between its daughter cells upon division. The model demonstrates that macrophage proliferation can reduce necrotic core formation by enhancing the capacity for necrotic lipid consumption. However, results suggest that proliferation can also be a double-edged sword because the reduction in necrotic core often comes at the expense of a substantially enlarged macrophage population.

Ford et al. (2019a) and Chambers et al. (2022) assume that all plaque macrophage behaviours occur at constant rates independent of internalised lipid content. In this paper, we generalise their lipid-structured framework to include live macrophage behaviour that depends smoothly and continuously on ingested lipid load. For now, we include lipid-dependent behaviour only in macrophage apoptosis, emigration and proliferation. Modelling of lipid-dependent phagocytosis and efferocytosis will be addressed in a future study. By simulating and analysing this new model, we demonstrate that lipid-dependent macrophage behaviour can substantially alter plaque fate by changing both the distribution and the net accumulation of lipid in the system. Note that, while lipid loading is believed to influence plaque fate by modulating the phenotypic profile of the macrophage population (Moore et al. 2013; Tabas and Bornfeldt 2016), we do not explicitly consider macrophage phenotype here. The current work can be regarded as a step towards more detailed mathematical models that link macrophage phenotype to internalised lipid load.

The remainder of the manuscript is structured as follows. In Sect. 2, we outline our methodology. This includes a brief presentation of the model equations, and definitions of the functions that characterise lipid-dependent macrophage behaviour. Section 3 reports results and analysis from an in-depth simulation study that addresses how lipid-dependent apoptosis, emigration and proliferation influence plaque progression. We discuss the implications of our results for both theoretical and experimental atherosclerosis research in Sect. 4, and end with broad conclusions on the significance of the study in Sect. 5.

## Methods

### Definitions

The number densities of live and apoptotic plaque macrophages with lipid load $a \geqslant a_0$ at time $t \geqslant 0$ are denoted $m\left( a,t\right)$ and $p\left( a,t\right)$, respectively. The minimum lipid load $a_0$ represents the endogenous lipid contained in the internal structures of each cell. We denote the acellular necrotic lipid content of the plaque at time *t* by $N\left( t\right)$. For $m\left( a,t\right)$ and $p\left( a,t\right)$, we define the total number of cells in each population by:

1 $$\begin{aligned} M\left( t\right) =\int _{a_0}^{\infty } m\left( a,t\right) da, \;\;\; P\left( t\right) =\int _{a_0}^{\infty } p\left( a,t\right) da, \end{aligned}$$

and the total lipid content of each population by:

2 $$\begin{aligned} A_M\left( t\right) =\int _{a_0}^{\infty } a m\left( a,t\right) da, \;\;\; A_P\left( t\right) =\int _{a_0}^{\infty } a p\left( a,t\right) da, \end{aligned}$$

respectively.

Lipid-dependent cell behaviour is modelled by assuming that the dimensional reference rate of a given behaviour is modulated by a dimensionless factor $g_\diamond \left( a\right)$. The symbol $\diamond$ is a placeholder that represents $\beta$ for apoptosis, $\gamma$ for emigration or $\rho$ for proliferation. For notational convenience, we define the related quantities:

3 $$\begin{aligned} G_\diamond \left( t\right) = \int _{a_0}^{\infty } g_\diamond \left( a\right) m\left( a,t\right) \, da, \;\;\; G_{\diamond a}\left( t\right) = \int _{a_0}^{\infty } g_\diamond \left( a\right) a m\left( a,t\right) \, da, \end{aligned}$$

which will appear in the model equations below. Lipid-independence in a given behaviour can be recovered by setting $g_\diamond \left( a\right) \equiv 1$. This, in turn, gives $G_\diamond \left( t\right) = M\left( t\right)$ and $G_{\diamond a}\left( t\right) = A_M\left( t\right)$.

### Model Statement

A formal derivation of the original lipid-independent model can be found in Ford et al. (2019a) and, for the macrophage proliferation terms, in Chambers et al. (2022). The interested reader is directed to these works for in-depth explanation. Here, we give a brief summary of the modelling assumptions before stating the equations in full. The model considers the following processes in the plaque:

1. Live macrophages consume lipid from LDL particles and offload lipid to HDL particles. Lipid attached to LDL particles is assumed to enter the plaque at constant rate $\lambda _L$ and be rapidly consumed by macrophages. A quasi-steady state approximation gives $\frac{\lambda _L}{M}$ as the rate per cell of lipid ingestion from LDL. HDL particles are assumed to enter the plaque with a constant lipid capacity per time $\lambda _H$ and rapidly fill with lipid offloaded from macrophages. A quasi-steady state approximation gives $\frac{\lambda _H}{M}$ as the rate per cell of lipid offloading to HDL. The *net* rate of lipid acquisition per cell from these two processes is $\frac{\lambda _L - \lambda _H}{M} = \frac{\lambda }{M}$. We assume that $\lambda > 0$ is necessary for a plaque to form. This continuous acquisition of lipid is modelled by an advection term in the $m\left( a,t\right)$ equation.
2. Live macrophages consume necrotic lipid at a rate proportional to $\theta$. This phagocytic lipid uptake is also modelled by continuous advection in the $m\left( a,t\right)$ equation.
3. Live macrophages become apoptotic macrophages at rate $\beta g_\beta \left( a\right)$ or emigrate from the plaque at rate $\gamma g_\gamma \left( a\right)$.
4. Live macrophages consume apoptotic macrophages by ingesting their entire lipid content at a rate proportional to $\eta$. This process (efferocytosis) contributes a local sink term and a non-local source term to the $m\left( a,t\right)$ equation. The source term takes the form of a convolution integral, which accounts for all possible consumption events that produce live macrophages with lipid content *a*. The value of this integral is interpreted as 0 for all $a \leqslant 2a_0$.
5. Live macrophages proliferate at rate $\rho g_\rho \left( a\right)$. Shortly before division, we assume that the parent cell newly-synthesises the quantity of endogenous lipid $a_0$ that is required to form a second daughter cell (Scaglia et al. 2014). Upon division, we assume that the total lipid content of the parent is divided equally between the two daughters. Proliferation contributes a local sink term and a non-local source term to the $m\left( a,t\right)$ equation. The source term accounts for the fact that daughter cells with lipid content *a* are produced by parent cells with lipid content $2a-a_0$. The transient, pre-division increase in the parent cell lipid content is not explicitly modelled as it occurs on a timescale much shorter than the timescale of interest.
6. Apoptotic macrophages undergo post-apoptotic necrosis, producing necrotic lipid at rate $\nu$.
7. Live macrophages with lipid content $a_0$ are recruited to the plaque from the bloodstream. The rate of cell recruitment is assumed to be a saturating function of the total ingested lipid in the live macrophage population $A_M\left( t\right) - a_0 M\left( t\right)$, with maximal recruitment rate $\alpha$ and half-maximal recruitment when $A_M\left( t\right) - a_0 M\left( t\right) = \kappa$. Note that this model for macrophage recruitment encodes an implicit lipid-dependence because the expression $A_M - a_0 M$ arises from an assumption that macrophages produce recruitment-stimulating cytokines at a rate proportional to their accumulated lipid content $a-a_0$. Recruitment is modelled by a boundary condition on the $m\left( a,t\right)$ equation.

The equations and boundary condition that reflect the above assumptions are as follows:

4 $$\begin{aligned}{} & {} \frac{\partial m\left( a,t\right) }{\partial t} + \bigg [\frac{\lambda }{M\left( t\right) } + \theta N\left( t\right) \bigg ] \, \frac{\partial m\left( a,t\right) }{\partial a} \nonumber \\{} & {} \quad = \eta \int _{a_0}^{a-a_0} m(a',t) p(a-a',t) \, da' + 4 \rho g_\rho \left( 2a-a_0\right) m(2a-a_0, t) \nonumber \\{} & {} \qquad - \Big [\beta g_\beta \left( a\right) + \gamma g_\gamma \left( a\right) + \rho g_\rho \left( a\right) + \eta P\left( t\right) \Big ] m\left( a,t\right) , \end{aligned}$$

5 $$\begin{aligned}{} & {} \frac{\partial p\left( a,t\right) }{\partial t} = \beta g_\beta \left( a\right) m\left( a,t\right) - \big [\nu M\left( t\right) + \eta \big ] p\left( a,t\right) , \end{aligned}$$

6 $$\begin{aligned}{} & {} \frac{dN\left( t\right) }{dt} = \nu A_P\left( t\right) - \theta M\left( t\right) N\left( t\right) , \end{aligned}$$

7 $$\begin{aligned}{} & {} \qquad \bigg [\frac{\lambda }{M\left( t\right) } + \theta N\left( t\right) \bigg ]m\left( a_0,t\right) = \frac{\alpha \big [A_M\left( t\right) - a_0 M\left( t\right) \big ]}{\kappa + A_M\left( t\right) - a_0 M\left( t\right) }. \end{aligned}$$

Additional ODEs for $M\left( t\right)$, $A_M\left( t\right)$, $P\left( t\right)$ and $A_P\left( t\right)$ can be generated by integrating (4) and (5) with respect to *a*, both with and without multiplying by *a* prior to the integration. For these calculations, we impose the requirement that $m\left( a,t\right)$, $a m\left( a,t\right)$, $p\left( a,t\right)$ and $a p\left( a,t\right)$ all $\rightarrow$ 0 as $a \rightarrow \infty$. This leads to the following equations:

8 $$\begin{aligned} \frac{dM\left( t\right) }{dt}= & {} \frac{\alpha \big [A_M\left( t\right) - a_0 M\left( t\right) \big ]}{\kappa + A_M\left( t\right) - a_0 M\left( t\right) } + \rho G_\rho \left( t\right) - \beta G_\beta \left( t\right) - \gamma G_\gamma \left( t\right) , \end{aligned}$$

9 $$\begin{aligned} \frac{dA_M\left( t\right) }{dt}= & {} \frac{a_0 \alpha \big [A_M\left( t\right) - a_0 M\left( t\right) \big ]}{\kappa + A_M\left( t\right) - a_0 M\left( t\right) } + \lambda + \theta M\left( t\right) N\left( t\right) \nonumber \\{} & {} + \eta A_P\left( t\right) + a_0 \rho G_\rho \left( t\right) - \beta G_{\beta a}\left( t\right) - \gamma G_{\gamma a}\left( t\right) , \end{aligned}$$

10 $$\begin{aligned} \frac{dP\left( t\right) }{dt}= & {} \beta G_\beta \left( t\right) - \big [\nu M\left( t\right) + \eta \big ] P\left( t\right) , \end{aligned}$$

11 $$\begin{aligned} \frac{dA_P\left( t\right) }{dt}= & {} \beta G_{\beta a}\left( t\right) - \big [\nu M\left( t\right) + \eta \big ] A_P\left( t\right) . \end{aligned}$$

Based on a similar argument to that presented in Chambers et al. (2022), we note that Eqs. (8) and (9) will lead to unbounded growth of $M\left( t\right)$ and $A_M\left( t\right)$ if:

$$\begin{aligned} \beta G_\beta \left( t\right) + \gamma G_\gamma \left( t\right) - \rho G_\rho \left( t\right) \leqslant 0. \end{aligned}$$

To avoid this eventuality in the current study, we ensure that the condition:

12 $$\begin{aligned} \beta g_\beta \left( a\right) + \gamma g_\gamma \left( a\right) - \rho g_\rho \left( a\right) > 0, \end{aligned}$$

is satisfied for all $a \in [a_0, \infty )$.

In the presence of lipid-dependent cell behaviour, the ODE equations (6), (8)–(11) and the PDE equations (4)–(5) cannot be readily decoupled, in contrast to the simpler models of Ford et al. (2019a) and Chambers et al. (2022). This limits the opportunity for analytical investigation of the model equations and, hence, this paper will use numerical simulations to study plaque fate and dynamics in a range of scenarios.

The model is closed by assigning appropriate initial conditions to each variable. The PDE variables require initial distributions and the ODE variables require initial values. Generically, we set:

13 $$\begin{aligned} \begin{aligned}&m\left( a,0\right) =m_0\left( a\right) , \; p\left( a,0\right) =p_0\left( a\right) , \\&M\left( 0\right) =M_0, \; P\left( 0\right) =P_0, \; A_M\left( 0\right) =A_{M0}, \; A_P\left( 0\right) =A_{P0}, \; N\left( 0\right) =N_0. \end{aligned} \end{aligned}$$

For $m_0\left( a\right)$ and $p_0\left( a\right)$, we use the following half-normal distributions:

14 $$\begin{aligned} \frac{m_0\left( a\right) }{M_0} = \frac{p_0\left( a\right) }{P_0} = \frac{2}{a_\sigma \sqrt{2\pi }} \, \exp \left( - \, \frac{\left( a \, - \, a_0\right) ^2}{2 {a_\sigma }^2}\right) , \end{aligned}$$

which are scaled such that $\int _{a_0}^{\infty } m_0\left( a\right) \, da = M_0$ and $\int _{a_0}^{\infty } p_0\left( a\right) \, da = P_0$. The parameter $a_\sigma > 0$ defines the shape of the distributions. For $A_{M0}$ and $A_{P0}$, we correspondingly define:

15 $$\begin{aligned} A_{M0} = M_0\left( a_0 \, + \, \frac{2}{\sqrt{2\pi }} \, a_\sigma \right) , \;\; A_{P0} = P_0\left( a_0 \, + \, \frac{2}{\sqrt{2\pi }} \, a_\sigma \right) . \end{aligned}$$

The initial number of live macrophages $M_0$ can be defined in terms of $a_\sigma$ by assuming that the initial distribution $m_0\left( a\right)$ satisfies the boundary condition (7) at $t = 0$. This leads to the relationship:

16 $$\begin{aligned} M_0 = \frac{\kappa \lambda \sqrt{2 \pi }}{a_\sigma \Big [a_\sigma \alpha \sqrt{2 \pi } - 2 \lambda \Big ]}. \end{aligned}$$

For a valid (positive) $M_0$ value, we require $a_\sigma > \frac{\lambda \sqrt{2}}{\alpha \sqrt{\pi }}$. Finally, we assume that initially there is no necrotic lipid in the system ($N_0 = 0$), and the system contains fewer dead cells than live cells. To satisfy the latter assumption, we arbitrarily set $P_0 = 0.5 M_0$.

### Lipid-Dependent Rate Functions

In the model, lipid-dependent cell behaviour is described by either a monotonic function $g_\diamond \left( a\right) = g_\diamond ^s\left( a\right)$ or a non-monotonic function $g_\diamond \left( a\right) = g_\diamond ^r\left( a\right)$. For the monotonic function, we use the following saturating relationship:

17 $$\begin{aligned} g_\diamond ^s\left( a\right) =\frac{\left( a_\diamond - a_0\right) ^{n_\diamond } + \delta _\diamond \left( a - a_0\right) ^{n_\diamond }}{\left( a_\diamond - a_0\right) ^{n_\diamond } + \left( a - a_0\right) ^{n_\diamond }}. \end{aligned}$$

Here, $g_\diamond ^s\left( a_0\right) = 1$ and the non-negative parameter $\delta _\diamond = \lim _{a\rightarrow \infty } g_\diamond ^s\left( a\right)$. Hence, $g_\diamond ^s\left( a\right)$ increases from 1 to $\delta _\diamond$ when $\delta _\diamond > 1$ or decreases from 1 to $\delta _\diamond$ when $0 \leqslant \delta _\diamond < 1$. The exponent $n_\diamond \geqslant 1$ determines the shape of the function and the parameter $a_\diamond > a_0$ denotes the lipid content for which $g_\diamond ^s\left( a_\diamond \right) = \frac{1 + \delta _\diamond }{2}$. For the non-monotonic function, we use the following scaled relationship:18where $0 \leqslant \epsilon _\diamond < 1$, $b_\diamond > 0$ and the exponents $q_\diamond$ and $k_\diamond$ satisfy $q_\diamond > k_\diamond \geqslant 1$. This function increases from $g_\diamond ^r\left( a_0\right) = \epsilon _\diamond$ to a peak value of 1 at , before decreasing towards $\epsilon _\diamond$ as *a* tends to infinity. The exponent $k_\diamond$ controls the rate of increase of $g_\diamond ^r\left( a\right)$ for $a \approx a_0$, while the larger exponent $q_\diamond$ controls the rate of decline of $g_\diamond ^r\left( a\right)$ as $a \rightarrow \infty$.

In practice, we shall primarily focus on modulating factors that have a monotonic dependence on *a* (i.e. monotonic increasing for $g_\beta \left( a\right)$, monotonic decreasing for $g_\gamma \left( a\right)$ and $g_\rho \left( a\right)$). However, for $g_\gamma \left( a\right)$, we shall also consider the non-monotonic dependence, since physical arguments suggest that this may be a more realistic assumption.

### Nondimensionalisation

Using tildes to denote dimensionless quantities, the independent and dependent variables are nondimensionalised as follows (Chambers et al. 2022):

19 $$\begin{aligned} {\tilde{a}}= & {} \frac{a}{a_0}, \; {\tilde{t}} = \beta t, \nonumber \\ {\tilde{m}}\left( {\tilde{a}},{\tilde{t}}\right)= & {} \frac{a_0}{M\left( t\right) } \, m\left( a,t\right) , \; {\tilde{p}}\left( {\tilde{a}},{\tilde{t}}\right) = \frac{a_0}{P\left( t\right) } \, p\left( a,t\right) , \nonumber \\ {\tilde{M}}\left( {\tilde{t}}\right)= & {} \frac{\beta }{\alpha } \, M\left( t\right) , \; {\tilde{P}}\left( {\tilde{t}}\right) = \frac{\beta }{\alpha } \, P\left( t\right) , \nonumber \\ {\tilde{A}}_M\left( {\tilde{t}}\right)= & {} \frac{\beta }{a_0 \, \alpha } \, A_M\left( t\right) , \; {\tilde{A}}_P\left( {\tilde{t}}\right) = \frac{\beta }{a_0 \, \alpha } \, A_P\left( t\right) , \; {\tilde{N}}\left( {\tilde{t}}\right) = \frac{\beta }{a_0 \, \alpha } \, N\left( t\right) . \end{aligned}$$

These scalings are chosen to give $\int _{1}^{\infty } {\tilde{m}}\left( {\tilde{a}},{\tilde{t}}\right) \, d{\tilde{a}} = \int _{1}^{\infty } {\tilde{p}}\left( {\tilde{a}},{\tilde{t}}\right) \, d{\tilde{a}} = 1$, such that ${\tilde{m}}\left( {\tilde{a}},{\tilde{t}}\right)$ and ${\tilde{p}}\left( {\tilde{a}},{\tilde{t}}\right)$ may be considered as probability density functions for the live and apoptotic macrophage populations, respectively. We further define the following dimensionless parameters:

20 $$\begin{aligned} {\tilde{\gamma }} = \frac{\gamma }{\beta }, \;\; {\tilde{\kappa }} = \frac{\kappa \, \beta }{a_0 \, \alpha }, \;\; {\tilde{\rho }} = \frac{\rho }{\beta }, \;\; {\tilde{\nu }} = \frac{\nu }{\beta }, \;\; {\tilde{\lambda }} = \frac{\lambda }{a_0 \, \alpha }, \;\; {\tilde{\theta }} = \frac{\theta \, \alpha }{\beta ^2}, \;\; {\tilde{\eta }} = \frac{\eta \, \alpha }{\beta ^2},\nonumber \\ {\tilde{a}}_\diamond = \frac{a_\diamond }{a_0}, \;\; {\tilde{b}}_\diamond = \frac{b_\diamond }{a_0}, \;\; {\tilde{a}}_\sigma = \frac{a_\sigma }{a_0}, \;\; {\tilde{n}}_\diamond = n_\diamond , \;\; {\tilde{\delta }}_\diamond = \delta _\diamond , \;\; {\tilde{k}}_\diamond = k_\diamond , \;\; {\tilde{q}}_\diamond = q_\diamond , \nonumber \\ {\tilde{M}}_0 = \frac{\beta }{\alpha } \, M_0, \;\; {\tilde{P}}_0 = \frac{\beta }{\alpha } \, P_0, \;\; {\tilde{A}}_{M0} = \frac{\beta }{a_0 \, \alpha } \, A_{M0}, \;\; {\tilde{A}}_{P0} = \frac{\beta }{a_0 \, \alpha } \, A_{P0}. \end{aligned}$$

The dimensionless model equations are presented in full in Appendix A. Appendix B presents three additional ODEs that describe the time evolution of the total amount of lipid in the system $L\left( t\right) = {\tilde{A}}_M\left( t\right) + {\tilde{A}}_P\left( t\right) + {\tilde{N}}\left( t\right)$, the average lipid content per live cell ${{\bar{A}}}_M\left( t\right) = \frac{{\tilde{A}}_M\left( t\right) }{{\tilde{M}}\left( t\right) }$, and the average lipid content per apoptotic cell ${{\bar{A}}}_P\left( t\right) = \frac{{\tilde{A}}_P\left( t\right) }{{\tilde{P}}\left( t\right) }$. These equations prove useful for interpreting the simulation outcomes. The numerical method used to solve the model equations is summarised in Appendix C.

### Base Case Model Parameterisation

The aim of this study is to investigate how lipid-dependent macrophage behaviour can alter the fate and dynamics of a plaque compared to cases where macrophage behaviour is lipid-independent. Thus, to maintain our focus on the role of lipid-dependence, we vary only the parameter values that characterise the functions $g_\diamond \left( a\right)$ and we fix all parameter values that are not related to these functions (see Table 1). Details on the parameterisation of the functions $g_\diamond \left( a\right)$ are provided at the beginning of Sect. 3. Here, we provide justification for the values of the lipid-independent base case parameters.

We estimate that $\beta = \nu = 0.05$ h$^{-1}$. The value for the apoptosis rate $\beta$ is consistent with estimates from Thon et al. (2018) and Ford et al. (2019b), who fitted models to data from *in vitro* experiments on macrophage lipid loading. The secondary necrosis rate $\nu$ is estimated from observations that cell lysis occurs between 12-24 h after apoptosis (Collins et al. 1997).

Using data from an *in vivo* study of the monocyte/macrophage inflammatory response in murine myocardial infarction, we estimate $\gamma = 0.01$ h$^{-1}$ and $\alpha = 10^4$ cells h$^{-1}$ (Leuschner et al. 2012). The value of the emigration rate $\gamma$ corresponds to the observation that 5–15% of cells exit the site of inflammation rather than die *in situ*, where we take our estimate at the upper end of the interval. The value for the maximal macrophage recruitment rate $\alpha$ is a conservative estimate based on the observation that the monocyte/macrophage turnover in the inflamed infarct exceeds $10^6$ cells in a 24 h period.

For the efferocytosis rate, we choose $\eta = 2.4 \times 10^{-6}$ cell$^{-1}$ h$^{-1}$, which is consistent with estimates made by Marée et al. (2005) in a study that fits ODE models to *in vitro* data on macrophage engulfment of apoptotic cells. An accurate estimate of the necrotic lipid ingestion rate $\theta$ is not currently available. However, it is believed that the rate of macrophage phagocytosis of apoptotic cells is considerably more efficient than that of necrotic material (Kojima et al. 2017). Hence, we assume that $\eta > \theta$, and set $\theta = 1.5 \times 10^{-7}$ cell$^{-1}$ h$^{-1}$.

Due to a lack of suitable quantitative data, estimates for the values of the remaining parameters $\rho$, $\lambda$ and $\kappa$ are not currently available. For macrophage proliferation, we consider the two cases $\rho = 0$ h$^{-1}$ and $\rho = 0.025$ h$^{-1}$. Here, the larger value ensures compliance with the condition (12), and, in the absence of other factors, predicts a biologically reasonable macrophage population doubling time of approximately 28 h. For $\lambda$ and $\kappa$, we choose dimensionless values that lead to a mild inflammatory response and relatively benign plaque formation in the absence of lipid-dependent effects. By making this choice, we leave scope to investigate whether lipid-dependent macrophage behaviour can, as one might anticipate, promote a more robust inflammatory response and lead to potentially dangerous plaque formation.

## Results

The model outlined in Sect. 2 includes lipid-dependent terms for macrophage apoptosis, emigration and proliferation. We stress, however, that it is not our intention to apply the model in its full generality. Rather, we shall consider lipid-dependence in each behaviour individually to investigate how each lipid-dependent behaviour can influence plaque progression. For the functions $g_\diamond \left( a\right)$, we consider a range of parameterisations, both in unscaled and scaled formats. We use a range of parameterisations for the lipid-dependent cell behaviours because there is a lack of appropriate experimental data with which to accurately estimate the relevant quantities. For unscaled simulations, we apply the functions $g_\diamond ^s\left( a\right)$ or $g_\diamond ^r\left( a\right)$ exactly as defined in Sect. 2.4. For scaled simulations, we pre-multiply the relevant function by a scaling value that we have found (by simulation) to give $G_\diamond \left( t\right) \approx 1$ at steady-state (specifically, we accept $G_\diamond \left( \infty \right) = 1 \pm 0.01$). Scaling the functions in this way allows for a consistent comparison of steady-state results from both lipid-dependent *and* lipid-independent cases because the net (population level) rate of the behaviour of interest is approximately conserved. We focus mainly on steady state results because the time to reach steady state (typically around 100 macrophage lifetimes) is considerably shorter than the lifespan of a plaque. Table 2 (monotonic functions) and Table 3 (non-monotonic functions) summarise the cases that we consider in the following sections. Each table reports function parameterisations, $G_\diamond \left( \infty \right)$ values from unscaled simulations and corresponding scaling values required to give $G_\diamond \left( \infty \right) \approx 1$.

**Table 1:** Base case lipid-independent parameter values

Parameter	Description	Value
$\gamma$	Reference dimensionless live cell emigration rate	0.2
$\kappa$	Dimensionless live cell accumulated lipid content for half-maximal recruitment	$\frac{25}{6}$
$\rho$	Reference dimensionless live cell proliferation rate	0 (Sect. 3.2)
		0.5 (Sect. 3.3)
$\nu$	Dimensionless post-apoptotic necrosis rate	1
$\lambda$	Dimensionless net LDL consumption/HDL offloading rate	0.1
$\theta$	Dimensionless necrotic lipid consumption rate	0.6
$\eta$	Dimensionless efferocytosis rate	9.6

These values are used for all simulation results in Sect. 3. The parameter $\rho$ has two possible values because we neglect proliferation for the results in Sect. 3.2, but include it for the results in Sect. 3.3

**Table 2:** Parameterisations, unscaled $G_\diamond \!\left( \infty \right)$ values and corresponding scaling values for simulations with the monotonic rate modulating function (32)

Cell behaviour	$g_\diamond \!\left( a\right)$	Parameter values			No proliferation ($\rho = 0$)		Proliferation ($\rho = 0.5$)	
		$a_\diamond$	$\delta _\diamond$	$n_\diamond$	$G_\diamond \!\left( \infty \right)$	Scaling Values	$G_\diamond \!\left( \infty \right)$	Scaling Values
					(unscaled)	($G_\diamond \!\left( \infty \right) \approx 1$)	(unscaled)	($G_\diamond \!\left( \infty \right) \approx 1$)
Apoptosis	$g_\beta ^s\left( a\right)$	15	2	2	1.188	0.86	Not considered	
Apoptosis	$g_\beta ^s\left( a\right)$	12	3	2	1.576	0.72	1.445	0.76
Apoptosis	$g_\beta ^s\left( a\right)$	9	4	2	2.367	0.565	Not considered	
Emigration	$g_\gamma ^s\left( a\right)$	12	0.1	1.5	0.6902	1.38*	Not considered	
Emigration	$g_\gamma ^s\left( a\right)$	18	0.1	1.5	0.7812	1.25*	0.7650	1.25*
Emigration	$g_\gamma ^s\left( a\right)$	24	0.1	1.5	0.8340	1.18*	Not considered	
Proliferation	$g_\rho ^s\left( a\right)$	4	0	2	Not applicable		0.6377	1.47
Proliferation	$g_\rho ^s\left( a\right)$	9	0	2	Not applicable		0.8070	1.22
Proliferation	$g_\rho ^s\left( a\right)$	14	0	2	Not applicable		0.8675	1.15

Asterisks denote that $\delta _\gamma$ is divided by the scaling value when the scaling is applied. This ensures that both the scaled and unscaled functions tend towards the same value as $a \rightarrow \infty$

**Table 3:** Parameterisations, unscaled $G_\diamond \!\left( \infty \right)$ values and corresponding scaling values for simulations with the non-monotonic rate modulating function (33)

Cell Behaviour	$g_\diamond \!\left( a\right)$	Parameter values				$G_\diamond \!\left( \infty \right)$	Scaling Values
		$\epsilon _\diamond$	$b_\diamond$	$k_\diamond$	$q_\diamond$	(unscaled)	($G_\diamond \!\left( \infty \right) \approx 1$)
Emigration	$g_\gamma ^r\left( a\right)$	0.1	3	1	2	0.5736	1.9*
Emigration	$g_\gamma ^r\left( a\right)$	0.1	6	1	2	0.5418	1.99*
Emigration	$g_\gamma ^r\left( a\right)$	0.1	9	1	2	0.4964	2.08*

Note that these simulations do not consider macrophage proliferation (i.e. $\rho = 0$). Asterisks denote that scaling values multiply only the second term on the right-hand side of (33). This ensures that both the scaled and unscaled functions tend towards $\epsilon _\gamma$ as $a \rightarrow \infty$

### Lipid-Independent Base Case Simulations

**Fig. 1:**
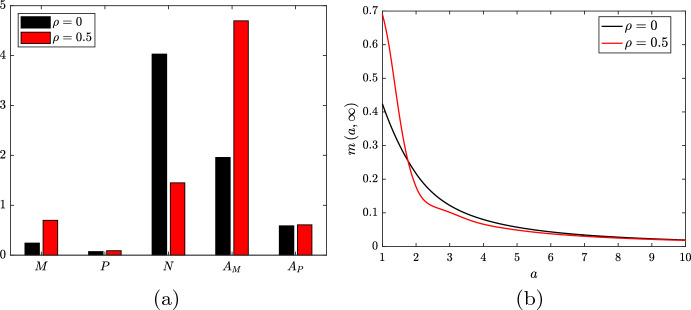
Steady state solutions for **a** the ODE variables and **b** the live cell distribution $m\left( a,t\right)$ from lipid-independent simulations both without macrophage proliferation ($\rho = 0$; black bars/lines) and with macrophage proliferation ($\rho = 0.5$; red bars/lines). The apoptotic cell distributions $p\left( a,\infty \right)$ are effectively identical to the corresponding $m\left( a,\infty \right)$ plots in each case (Color figure online)

Intra-plaque macrophage proliferation has only recently been established as an important contributor to plaque progression (Robbins et al. 2013). The extent of this proliferation is not well characterised but it is understood to vary over the lifetime of a plaque (Lhoták et al. 2016). Given this uncertainty, we shall perform numerical investigations of lipid-dependent cell behaviour both in the absence (Sect. 3.2) and the presence (Sect. 3.3) of macrophage proliferation. To provide a reference point for our lipid-dependent simulations, we first generate base case results where macrophage behaviour is independent of internalised lipid (all $g_\diamond \left( a\right) = 1$). Steady-state results for these cases, which use only the parameter values in Table 1, are shown in Fig. 1. For an in-depth understanding of these results, interested readers are referred to the simulations and analysis in Ford et al. (2019a) and Chambers et al. (2022). Here, we provide only a brief summary of some key features of the results.

In the absence of proliferation ($\rho = 0$), the model recruits a relatively small macrophage population and forms a moderately sized necrotic core ($M \approx 0.24$, $N \approx 4.03$; Fig. 1a black bars). This reflects our conservative model parameterisation, since we wish to allow for elevated macrophage recruitment in lipid-dependent cases where poorer outcomes are anticipated (e.g. when apoptosis increases with *a*, or when emigration decreases with *a*). In the case with proliferation ($\rho = 0.5$), we observe an almost 3-fold increase in the live cell population ($M \approx 0.70$; Fig. 1a red bar). This is partly due to proliferation itself, but also due to enhanced recruitment courtesy of the substantial increase in $A_M$ (4.70 vs. 1.96). The increased cell population leads to a reduction in both the necrotic core size ($N \approx 1.45$) and the average lipid per cell (${{\bar{A}}}_M = {{\bar{A}}}_P \approx 6.7$, down from 8.1). The reduction in ${{\bar{A}}}_M$ due to proliferation can be seen qualitatively by comparing the steady state $m\left( a,t\right)$ distributions in Fig. 1b. The case with proliferation (red line) has a considerably larger proportion of cells with very small lipid loads.

For the lipid-independent parameterisation chosen here, there is no single macrophage behaviour that dominates plaque formation. Given that these lipid-independent parameters are fixed for the entirety of the study, we argue that avoiding a single dominant cell behaviour is the most appropriate way to gain a general appreciation of the impact of lipid-dependence on plaque fate and dynamics.

### Lipid-Dependent Simulations Without Proliferation

In this section, we neglect macrophage proliferation and investigate, in turn, the role of lipid-dependence in macrophage apoptosis and emigration.

#### Apoptosis Only

**Fig. 2:**
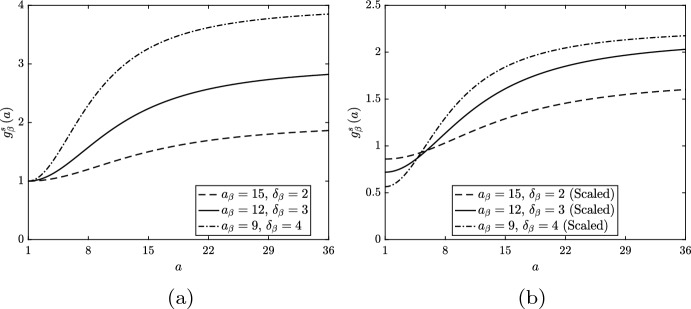
**a** Unscaled and **b** scaled rate modulating functions for macrophage apoptosis $g_\beta \left( a\right) = g_\beta ^s\left( a\right)$. Plots correspond to equation (32) with parameter values $n_\beta = 2$ and $a_\beta = 15$, $\delta _\beta = 2$ (dashed lines), $a_\beta = 12$, $\delta _\beta = 3$ (solid lines) or $a_\beta = 9$, $\delta _\beta = 4$ (dot-dashed lines). Scaling values for the plots in (**b**) are 0.86, 0.72 and 0.565, respectively

To investigate lipid-dependent apoptosis, we set $g_\gamma \left( a\right) = 1$ and $g_\beta \left( a\right) = g_\beta ^s\left( a\right)$. We consider three different unscaled forms for $g_\beta ^s$, each of which has $\delta _\beta > 1$ and $n_\beta = 2$ (Fig. 2a). Each function reflects an assumption that the likelihood of macrophage apoptosis increases with increasing ingested lipid content (Tabas 2002; Feng et al. 2003). By varying the values of both $a_\beta$ and $\delta _\beta$, we investigate the impact of a mild ($a_\beta = 15$, $\delta _\beta = 2$), moderate ($a_\beta = 12$, $\delta _\beta = 3$) or severe ($a_\beta = 9$, $\delta _\beta = 4$) increase in the apoptosis rate above the reference value with increasing *a*. (By changing the parameter values in this way, we anticipate that *all* scenarios with $a_\beta \in \left[ 9, 15\right]$ and $\delta _\beta \in \left[ 2, 4\right]$ will have solutions that lie within the bounds of those reported below.)

**Fig. 3:**
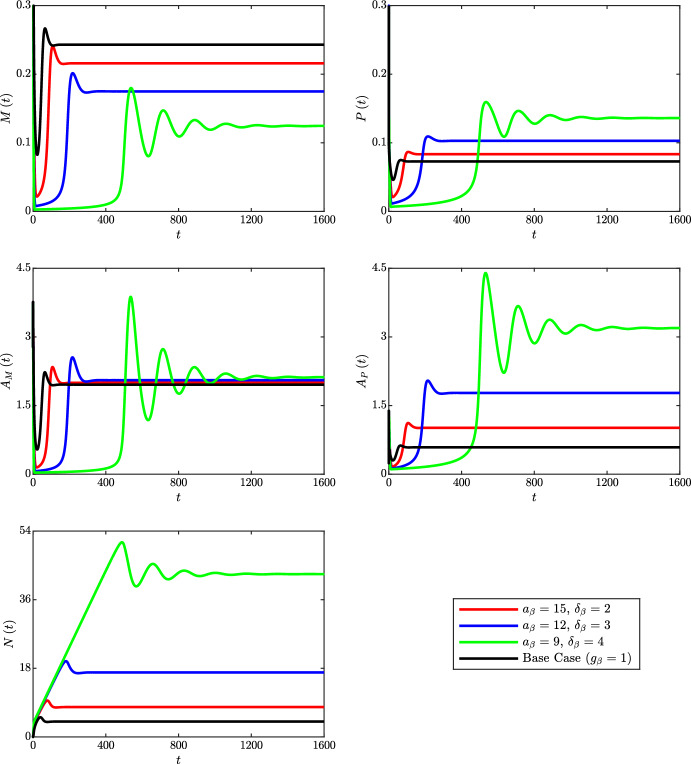
Time dependent solutions of the ODE variables $M\left( t\right)$, $P\left( t\right)$, $A_M\left( t\right)$, $A_P\left( t\right)$ and $N\left( t\right)$ for the lipid-independent base case ($g_\beta = 1$; black lines) and for three cases with unscaled lipid-dependent apoptosis ($g_\beta = g_\beta ^s$). Lipid-dependent cases have parameter values $n_\beta = 2$ and $a_\beta = 15$, $\delta _\beta = 2$ (red lines), $a_\beta = 12$, $\delta _\beta = 3$ (blue lines) or $a_\beta = 9$, $\delta _\beta = 4$ (green lines) (Color figure online)

**Fig. 4:**
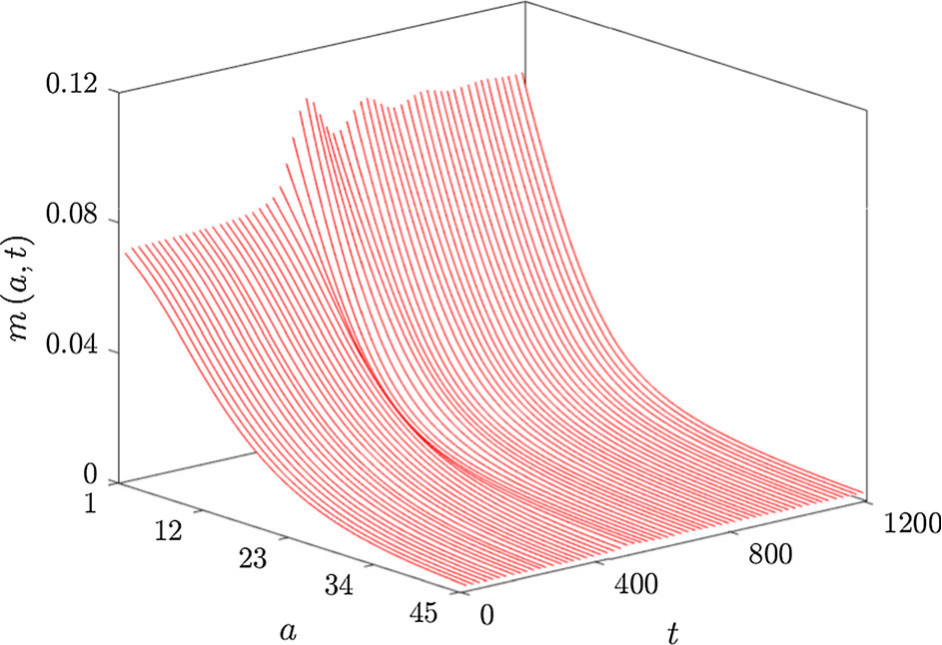
Surface plot showing the temporal evolution of $m\left( a,t\right)$ for $1 \leqslant a \leqslant 45$ and $0 \leqslant t \leqslant 1200$ in a simulation with unscaled lipid-dependent apoptosis ($g_\beta = g_\beta ^s$ with $n_\beta = 2$, $a_\beta = 9$ and $\delta _\beta = 4$). Note the onset of temporal oscillations in $m\left( a,t\right)$ near $a=1$ at $t \approx 400$. These oscillations reflect repeated bursts of macrophage recruitment that gradually diminish in intensity

Time dependent solutions of the ODE variables for these cases are compared with those for the base case simulation ($g_\beta \left( a\right) = 1$) in Fig. 3. An immediate observation is the interesting behaviour of the case with $a_\beta = 9$ and $\delta _\beta = 4$. While the other cases all display similar dynamics, this case elicits oscillations that eventually decay towards steady state. Noting that this is the case with the highest net apoptosis rate (dimensionless value $G_\beta \left( t\right) = 2.367$ at steady state; see Table 2), a likely explanation for the oscillations is as follows. Initially, live macrophage numbers $M\left( t\right)$ drop to a very low level because cells that ingest even moderate quantities of lipid quickly die to become apoptotic macrophages $P\left( t\right)$. The associated conversion of live cell lipid $A_M\left( t\right)$ to apoptotic cell lipid $A_P\left( t\right)$ reduces the rate of macrophage recruitment. Low cell numbers and sustained apoptotic lipid generation lead to rapid accumulation of necrotic lipid $N\left( t\right)$. Eventually, however, the necrotic lipid pool becomes so vast that the live macrophages begin to ingest lipid at a rate that outstrips the death rate. This allows $A_M\left( t\right)$ to rise, which stimulates macrophage recruitment and enhances lipid ingestion to shrink the necrotic core. Of course, as more live cells now attain higher quantities of ingested lipid, their apoptosis rates increase further and $M\left( t\right)$ drops once again. A new period of growth in $N\left( t\right)$ is then initiated until a further (smaller) wave of macrophage recruitment is triggered. This cycle repeats until the magnitude of the oscillations tend to zero. Figure 4 presents a corresponding surface plot of the live macrophage distribution $m\left( a,t\right)$ in this case. The solution shows temporal oscillations near $a = 1$, which are associated with the repeated waves of macrophage recruitment.

**Fig. 5:**
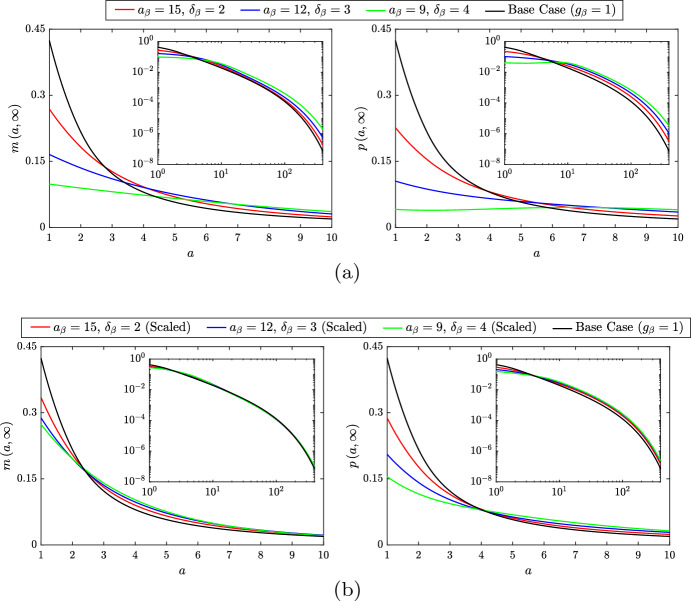
Steady state $m\left( a,t\right)$ distributions (left panels) and $p\left( a,t\right)$ distributions (right panels) for the lipid-independent base case ($g_\beta = 1$; black lines) and for three cases with **a** unscaled or **b** scaled lipid-dependent apoptosis ($g_\beta = g_\beta ^s$). Lipid-dependent cases have parameter values $n_\beta = 2$ and $a_\beta = 15$, $\delta _\beta = 2$ (red lines), $a_\beta = 12$, $\delta _\beta = 3$ (blue lines) or $a_\beta = 9$, $\delta _\beta = 4$ (green lines). Primary plots show the results on the interval $a \in \left[ 1,10\right]$ and inset log–log plots show the results on the entire *a* domain (Color figure online)

Long-time solutions of the ODE variables for each simulation indicate that the magnitude and steepness of the increase in $g_\beta ^s$ correlates with a decrease in *M* and an increase in each of the other variables (ranging from a marginal rise in $A_M$ to a substantial rise in *N*). The magnitude and steepness of the change in $g_\beta ^s$ also appears to be correlated to the time required to reach steady state, which increases from $t \approx 100$ in the base case to $t \approx 180$ ($a_\beta = 15$, $\delta _\beta = 2$), $t \approx 350$ ($a_\beta = 12$, $\delta _\beta = 3$) or $t \approx 2000$ ($a_\beta = 9$, $\delta _\beta = 4$). The steady state distributions of live macrophages $m\left( a,t\right)$ and apoptotic macrophages $p\left( a,t\right)$ for each simulation are presented in Fig. 5a (note, from Eq. (24), that $m\left( a,t\right)$ and $p\left( a,t\right)$ differ at steady state only in the case of lipid-dependent apoptosis). For both *m* and *p*, we see that increasing the magnitude and steepness of the change in $g_\beta ^s$ skews the distributions towards larger lipid loads. This seems counter-intuitive, but it appears that the elevated lipid ingestion rate (due to the increase in *N* and $A_P$) produces more cells with large lipid loads than are lost through apoptosis. At steady state, the *m* and *p* distributions are related by the expression $p\left( a,\infty \right) = \frac{g_\beta \left( a\right) m\left( a,\infty \right) }{G_\beta \left( \infty \right) }$ [see Eq. (24)]. In the simulation with $a_\beta = 9$ and $\delta _\beta = 4$, this gives a non-monotonic profile for $p\left( a,\infty \right)$ with a shallow peak near $a = 8$.

**Fig. 6:**
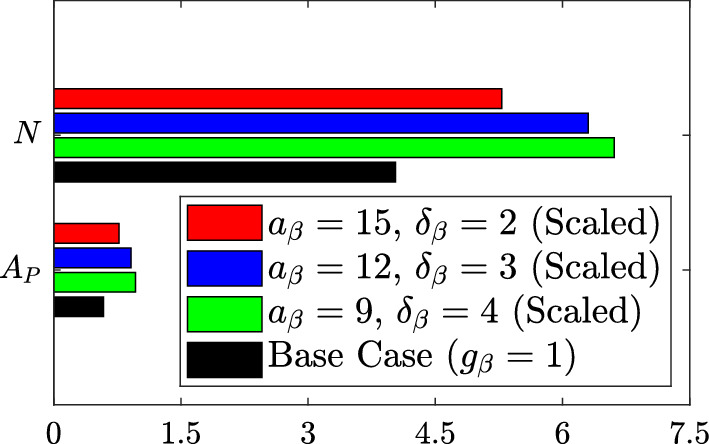
Steady state solutions of the ODE variables $A_P\left( t\right)$ and $N\left( t\right)$ for the lipid-independent base case ($g_\beta = 1$; black bars) and for three cases with scaled lipid-dependent apoptosis ($g_\beta = g_\beta ^s$). Lipid-dependent cases have parameter values $n_\beta = 2$ and $a_\beta = 15$, $\delta _\beta = 2$ (red bars), $a_\beta = 12$, $\delta _\beta = 3$ (blue bars) or $a_\beta = 9$, $\delta _\beta = 4$ (green bars). Solutions for $M\left( t\right)$, $P\left( t\right)$ and $A_M\left( t\right)$ are omitted from the plot as their values are unchanged across cases (Color figure online)

When interpreting these results, it is important to remember that the net apoptosis rates $G_\beta \left( t\right)$ vary considerably between the different simulations (from 1 in the base case to 2.367 at steady state in the case with $a_\beta = 9$ and $\delta _\beta = 4$). To correct for this difference, we repeat the lipid-dependent simulations with appropriately scaled functions $g_\beta ^s\left( a\right)$ that give $G_\beta \left( t\right) \approx 1$ at steady state (Fig. 2b). For these simulations, we find that the steady state values of *M*, *P* and $A_M$ effectively remain fixed, and only $A_P$ and *N* vary across the different cases. (The oscillatory dynamics observed above do not occur in this case, and, while the trend of increasing time to steady state remains, it is much less pronounced than before). Figure 6 compares the steady state solutions for $A_P$ and *N* from the base case with those from the scaled lipid-dependent simulations. While the absolute variation in these quantities is much smaller than in the unscaled scenario, the trend of increasing $A_P$ and *N* with increasing magnitude and steepness of the change in $g_\beta ^s$ remains.

Plots of the corresponding steady state PDE solutions are shown in Fig. 5b. These demonstrate that the trends in the $m\left( a,\infty \right)$ and $p\left( a,\infty \right)$ distributions with scaled $g_\beta ^s\left( a\right)$ are largely conserved from the unscaled cases (Fig. 5a), although the differences relative to the base case results are less pronounced. Each of the $p\left( a,\infty \right)$ distributions presented in Fig. 5b satisfies the relationship $p\left( a,\infty \right) = g_\beta \left( a\right) m\left( a,\infty \right)$. Accordingly, the $p\left( a,\infty \right)$ distribution in each case corresponds exactly to the distribution of apoptosis events at steady state. Moreover, Eq. (36) shows that, in each case, the steady state average lipid per apoptotic cell ${{\bar{A}}}_P = \frac{A_P}{P}$ is given by the steady state $G_{\beta a}$ value. Given that the steady state *P* values are essentially identical across all cases, this indicates that the steady state $A_P$ values are exactly proportional to the steady state $G_{\beta a}$ values. The trend observed in Fig. 6 for increasing steady state $A_P$ (and *N*) with increasing magnitude and steepness of the change in $g_\beta ^s$ is therefore related to a trend for increasing steady state $G_{\beta a}$. The precise reason for this trend of increasing steady state $G_{\beta a}$ is, however, not entirely clear. It may be correlated to the increase in the (maximum) steepness of $g_\beta ^s$, or it may be correlated to the increase in the limiting value of $g_\beta ^s$ (c.f. Fig. 2b).

The fact that the steady state *M* and $A_M$ values are essentially identical across these scaled simulations has interesting consequences when viewed through the lens of Eqs. (34) and (35). For the scenarios simulated here, Eq. (34) reduces to:

21 $$\begin{aligned} \frac{dL\left( t\right) }{dt} = F\left( t\right) + \lambda - \gamma A_M\left( t\right) . \end{aligned}$$

**Fig. 7:**
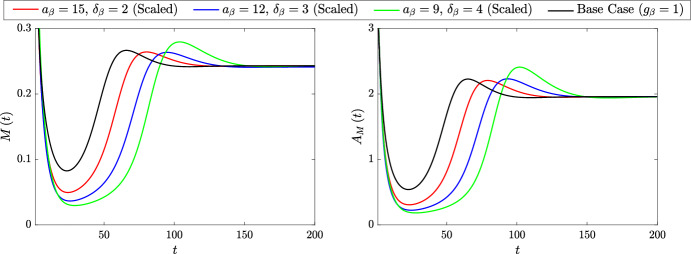
Time dependent solutions of the ODE variables $M\left( t\right)$ and $A_M\left( t\right)$ for the lipid-independent base case ($g_\beta = 1$; black lines) and for three cases with scaled lipid-dependent apoptosis ($g_\beta = g_\beta ^s$). Lipid-dependent cases have parameter values $n_\beta = 2$ and $a_\beta = 15$, $\delta _\beta = 2$ (red lines), $a_\beta = 12$, $\delta _\beta = 3$ (blue lines) or $a_\beta = 9$, $\delta _\beta = 4$ (green lines). Although all simulations reach similar steady state *M* and $A_M$ values, the paths taken to get there vary and this has consequences for the total quantity of lipid retained in the system (Color figure online)

The total system lipid $L\left( t\right)$ has explicit dependence only on $M\left( t\right)$ and $A_M\left( t\right)$. Interestingly, however, our simulation results indicate that the steady state *L* value is not uniquely determined by the steady state *M* and $A_M$ values (i.e. even though steady state *M* and $A_M$ are essentially fixed across simulations, there are differences in steady state *L* due to the variation in steady state $A_P$ and *N*). This observation demonstrates that, in terms of total system lipid, the model plaque carries the weight of its history. That is, the observed increase in steady state *L* with decreasing $a_\beta$ and increasing $\delta _\beta$ can be explained by the historical accumulation of lipid due to periods of reduced lipid removal by emigration (i.e. reduced $A_M\left( t\right)$) or increased lipid addition by recruitment (i.e. increased $F\left( t\right)$ or, equivalently, increased $A_M\left( t\right) - M\left( t\right)$). The time courses of the relevant $M\left( t\right)$ and $A_M\left( t\right)$ solutions (Fig. 7) suggest that reduced lipid removal by emigration is the predominant mechanism in this case.

Unlike $L\left( t\right)$, the steady state solution for ${{\bar{A}}}_M\left( t\right)$ remains fixed across all four simulations. In each of these cases, the steady state solution of Eq. (35) can be expressed as:

22 $$\begin{aligned} \frac{ \lambda }{M} + \theta N + \eta A_P - \frac{F}{M} \Big [{{\bar{A}}}_M - 1\,\Big ] + {{\bar{A}}}_M - G_{\beta a} = 0, \end{aligned}$$

where all time-dependent variables take their steady state values. As steady state *M* and ${{\bar{A}}}_M$ have fixed values for all simulations, only terms two, three and six on the left-hand side of (22) change in each case. Thus, steady state ${{\bar{A}}}_M$ is unchanged across all scaled simulations because any reduction in average lipid per cell due to lipid-dependent apoptosis (term six) is exactly compensated by an increase in necrotic and efferocytic lipid consumption (terms two and three, respectively). This finding supports our earlier interpretation of the unscaled simulation results, where we found that the direct contribution of lipid-dependent apoptosis to reducing ${{\bar{A}}}_M\left( t\right)$ was counteracted by other factors.

#### Emigration Only

To study lipid-dependent emigration, we set $g_\beta \left( a\right) = 1$ and consider that $g_\gamma \left( a\right)$ takes either the monotonic form $g_\gamma ^s\left( a\right)$ with $\delta _\gamma < 1$, or the non-monotonic form $g_\gamma ^r\left( a\right)$. In the monotonic case, we assume that the rate at which macrophages leave the plaque decreases with increasing lipid load (van Gils et al. 2012; Wanschel et al. 2013; Chen et al. 2019). In the non-monotonic case, we retain this argument. However, we additionally assume a substantially reduced emigration rate for macrophages with little or no accumulated lipid. This assumption reflects a range of considerations, including the innate propensity for macrophages to pursue foreign bodies, and the fact that emigrating macrophages typically traverse the plaque before exiting to the media (Llodrá et al. 2004). Either way, we anticipate that newly-recruited macrophages are unlikely to leave the plaque without first ingesting at least some lipid. Combined with the assumption of reduced emigration for macrophages with large lipid loads, this leads us to consider that the lipid-dependent emigration rate may peak at some intermediate lipid quantity. Note, however, that the argument regarding low rates of emigration for macrophages with small lipid loads may not hold if the lipid load can be reduced by proliferation. As such, we will only consider the function $g_\gamma ^r\left( a\right)$ for the model without macrophage proliferation.

**Fig. 8:**
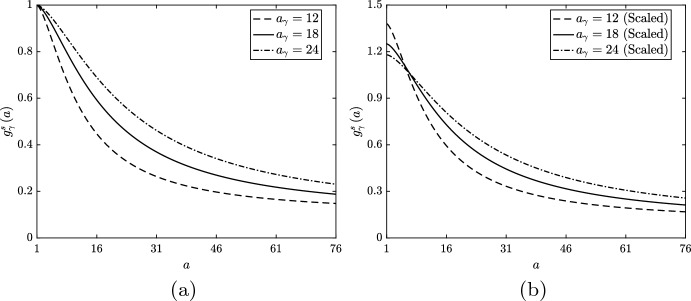
**a** Unscaled and **b** scaled rate modulating functions for macrophage emigration $g_\gamma \left( a\right) = g_\gamma ^s\left( a\right)$. Plots correspond to Eq. (32) with parameter values $n_\gamma = 1.5$, $\delta _\gamma = 0.1$ and $a_\gamma = 12$ (dashed lines), $a_\gamma = 18$ (solid lines) or $a_\gamma = 24$ (dot-dashed lines). Scaling values for the plots in (**b**) are 1.38, 1.25 and 1.18, respectively. In the scaled cases, $\delta _\gamma$ is divided by the scaling value so that each $g_\gamma ^s$ retains the limiting value of the unscaled functions

We first investigate monotonic lipid-dependence in macrophage emigration and consider three alternative unscaled forms for $g_\gamma ^s\left( a\right)$ (Fig. 8a). Each function has $\delta _\gamma = 0.1$ and $n_\gamma = 1.5$, while we vary $a_\gamma$ to study the impact of a gentle ($a_\gamma = 24$), moderate ($a_\gamma = 18$) or steep ($a_\gamma = 12$) reduction in the emigration rate as *a* increases over low values. We deliberately choose a small $n_\gamma$ value and non-zero $\delta _\gamma$ to maintain feasibility in the numerical solution of the equations. When the functions $g_\gamma ^s$ decline rapidly and/or tend towards zero as $a \rightarrow \infty$, a non-negligible proportion of cells can acquire extremely large lipid loads ($a \gg 1000$). For numerical accuracy, such cases need to be solved on extended domains at significant computational expense. We avoid such scenarios here, as this increase in computational effort is unlikely to produce additional physical insight. While physically we may expect effectively zero emigration of macrophages with very large lipid loads, our chosen parameterisation ensures that cells are still fifty times more likely to die than to emigrate as $a \rightarrow \infty$.

**Fig. 9:**
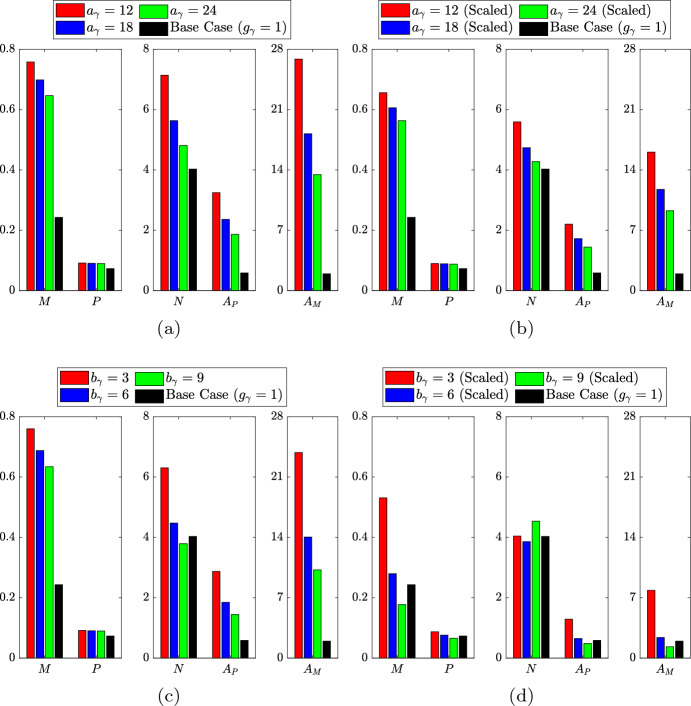
Steady state solutions of the ODE variables $M\left( t\right)$, $P\left( t\right)$, $N\left( t\right)$, $A_P\left( t\right)$ and $A_M\left( t\right)$ for the lipid-independent base case ($g_\gamma = 1$; black bars) and for several scenarios with lipid-dependent emigration. Lipid-dependent simulations use **a** unscaled or **b** scaled monotonic lipid-dependence ($g_\gamma = g_\gamma ^s$) and **c** unscaled or **d** scaled non-monotonic lipid-dependence ($g_\gamma = g_\gamma ^r$). Monotonic lipid-dependent cases have parameter values $n_\gamma = 1.5$, $\delta _\gamma = 0.1$ and $a_\gamma = 12$ (red bars), $a_\gamma = 18$ (blue bars) or $a_\gamma = 24$ (green bars). Non-monotonic lipid-dependent cases have parameter values $\epsilon _\gamma = 0.1$, $k_\gamma = 1$, $q_\gamma = 2$ and $b_\gamma = 3$ (red bars), $b_\gamma = 6$ (blue bars) or $b_\gamma = 9$ (green bars) (Color figure online)

**Fig. 10:**
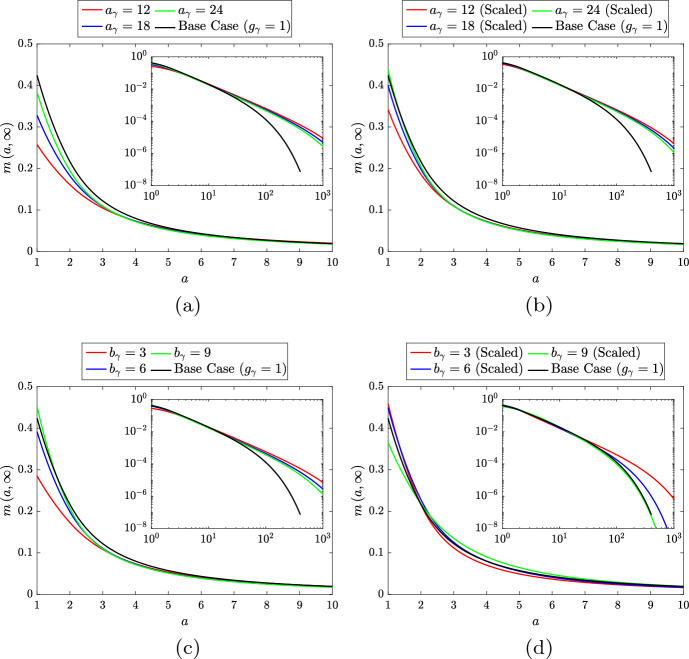
Steady state $m\left( a,t\right)$ distributions for the lipid-independent base case ($g_\gamma = 1$; black lines) and for several scenarios with lipid-dependent emigration. Lipid-dependent simulations use **a** unscaled or **b** scaled monotonic lipid-dependence ($g_\gamma = g_\gamma ^s$) and **c** unscaled or **d** scaled non-monotonic lipid-dependence ($g_\gamma = g_\gamma ^r$). Monotonic lipid-dependent cases have parameter values $n_\gamma = 1.5$, $\delta _\gamma = 0.1$ and $a_\gamma = 12$ (red lines), $a_\gamma = 18$ (blue lines) or $a_\gamma = 24$ (green lines). Non-monotonic lipid-dependent cases have parameter values $\epsilon _\gamma = 0.1$, $k_\gamma = 1$, $q_\gamma = 2$ and $b_\gamma = 3$ (red lines), $b_\gamma = 6$ (blue lines) or $b_\gamma = 9$ (green lines). Primary plots show the results on the interval $a \in \left[ 1,10\right]$ and inset log–log plots show the results on the entire *a* domain (Color figure online)

Steady state results for the ODE variables in each lipid-dependent case are compared with those from the base case ($g_\gamma = 1$) in Fig. 9a (note the use of several vertical axis scales for ease of visualisation). These results show all five variables trending upwards with decreasing $a_\gamma$ (or, equivalently, with decreasing net emigration rate $G_\gamma \left( \infty \right)$). These upward trends are of varying degrees, with the case for $a_\gamma = 12$ showing approximately 1.3-, 1.8-, 3.1-, 5.5- and 13.7-fold increases in *P*, *N*, *M*, $A_P$ and $A_M$, respectively, versus the base case. The increase in steady state *M* in each lipid-dependent case is partly due to reduced net emigration, but mainly due to increased cell recruitment as a consequence of the substantial rise in $A_M$. The steady state $A_M$ values increase so significantly because, as cells accumulate more and more lipid, they become less likely to emigrate. This is shown in the inset of Fig. 10a by the (relatively) large proportions of cells with very large lipid loads in the steady state $m\left( a,t\right)$ distributions. As these heavily lipid-loaded cells are much more likely to undergo apoptosis than to emigrate, $A_P$ is increased and this ultimately fuels an increase in *N*. These enlarged necrotic lipid pools emerge despite the significant increase in overall lipid consumption afforded by the increase in *M*.

To correct for the variation in the net steady state emigration rates in the above lipid-dependent cases, we repeat our study with appropriately scaled $g_\gamma ^s\left( a\right)$ functions. The functions are scaled in such a way that the limiting value of each $g_\gamma ^s$ remains at 0.1. In practice, this involves dividing $\delta _\gamma$ by the scaling value when the scaling is applied. We make this assumption because the model results are particularly sensitive to the limiting value of $g_\gamma ^s$ and we wish to retain consistency for comparison with the unscaled cases. Steady state solutions of the ODE variables are presented in Fig. 9b. Unlike lipid-dependent apoptosis, where the steady state values of *M*, *P* and $A_M$ were all unchanged from their base case values upon scaling, here we see that all five variables are different from the base case. Indeed, the trends in the ODE solutions are identical to those in the unscaled cases, although with slightly smaller variation from the base case values. This observation correlates with the steady state $m\left( a,t\right)$ distributions in Fig. 10b, which become slightly closer to the base case distribution than those in the unscaled case (Fig. 10a). These results with scaled $g_\gamma ^s$ demonstrate that the particular form of the lipid-dependent emigration function (parameterised here by $a_\gamma$ and an appropriate scaling value) can have considerable influence on the long-term plaque composition.

**Fig. 11:**
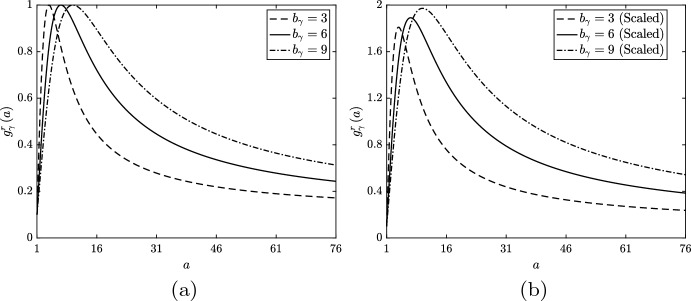
**a** Unscaled and **b** scaled rate modulating functions for macrophage emigration $g_\gamma \left( a\right) = g_\gamma ^r\left( a\right)$. Plots correspond to Eq. (33) with parameter values $\epsilon _\gamma = 0.1$, $k_\gamma = 1$, $q_\gamma = 2$ and $b_\gamma = 3$ (dashed lines), $b_\gamma = 6$ (solid lines) or $b_\gamma = 9$ (dot-dashed lines). Scaling values for the plots in (**b**) are 1.9, 1.99 and 2.08, respectively. These scaling values multiply only the second term in Eq. (33) so that each $g_\gamma ^r$ retains the limiting value of the unscaled functions

**Fig. 12:**
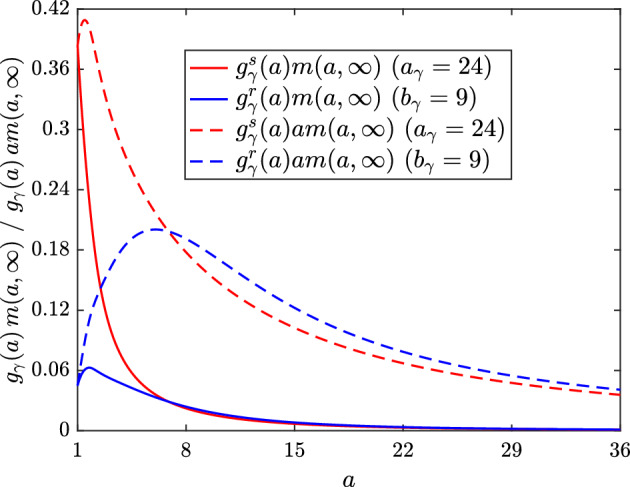
Plot comparing how emigration events $g_\gamma \left( a\right) m\left( a,\infty \right)$ (solid lines) and associated quantities of removed lipid $g_\gamma \left( a\right) am\left( a,\infty \right)$ (dashed lines) are distributed with respect to *a* at steady state in simulations with monotonic lipid-dependent emigration $g_\gamma = g_\gamma ^s$ ($a_\gamma = 24$; red lines) and non-monotonic lipid-dependent emigration $g_\gamma = g_\gamma ^r$ ($b_\gamma = 9$; blue lines). The net emigration rate for the non-monotonic case is considerably smaller than that for the monotonic case ($G_\gamma = \int _1^\infty g_\gamma m \, da \approx 0.50$ vs. 0.83), but the increased proportion of emigration events with larger lipid loads produces a similar net lipid removal rate ($G_{\gamma a} = \int _1^\infty g_\gamma am \, da \approx 6.29$ vs. 6.61). The average lipid removed per cell $\frac{G_{\gamma a}}{G_\gamma }$ is approximately 8.0 for the monotonic case and approximately 12.6 for the non-monotonic case

We now consider the case of non-monotonic lipid-dependent emigration, where the function $g_\gamma ^r\left( a\right)$ encodes a reduced emigration rate for macrophages with small lipid loads. We fix the parameter values $k_\gamma = 1$, $q_\gamma = 2$ and $\epsilon _\gamma = 0.1$ (note the consistency in the limiting value of $g_\gamma$), and vary $b_\gamma$ such that $g_\gamma ^r$ has its peak at $a = 4$ ($b_\gamma = 3$), $a = 7$ ($b_\gamma = 6$) or $a = 10$ ($b_\gamma = 9$). Plots of these three unscaled $g_\gamma ^r$ are shown in Fig. 11a, where we see that each rightward shift in the peak reduces the rate of decline of the function with increasing *a*. The steady state ODE solutions and $m\left( a,t\right)$ distributions generated with these functions are shown in Figs. 9c and 10c, respectively. An immediate observation is that the plot of the ODE results is visually very similar to that for the unscaled monotonic case (Fig. 9a). This, however, is coincidental and should not be regarded as significant. Indeed, the lipid-dependent case with the best outcome (smallest *N*) in Fig. 9a is the one with the highest net emigration rate at steady state ($a_\gamma = 24$, $G_\gamma \left( \infty \right) \approx 0.83$), whereas the lipid-dependent case with the best outcome in Fig. 9c is the one with the *lowest* net emigration rate at steady state ($b_\gamma = 9$, $G_\gamma \left( \infty \right) \approx 0.50$). This observation demonstrates that when emigration events are skewed towards macrophages with larger lipid loads, the efficiency of lipid removal from the system can be substantially improved (Fig. 12). The case with $b_\gamma = 9$ has a smaller steady state *N* value than the base case (where $G_\gamma = 1$), which presumably reflects a preferential balance in the steady state *M* and $A_P$ values. However, the exact mechanism that underlies this result is difficult to ascertain.

Given that non-monotonic lipid-dependent emigration can remove substantial amounts of lipid from the plaque even at reduced net emigration rates, it seems reasonable to expect an improvement in outcomes when the net emigration rate is scaled to the reference value. (See Fig. 11b for plots of the scaled lipid-dependent functions $g_\gamma ^r$. We again preserve the limiting value of 0.1 by multiplying only the non-constant part of (33) by the scaling value.) This is indeed true in some cases, but the steady state ODE results in Fig. 9d portray a greater subtlety in the model’s response to this scaling. Relative to their corresponding unscaled cases (Fig. 9c), each scaled case shows reduced steady state *M*, *P*, $A_M$ and $A_P$ values. Steady state *N* also decreases for $b_\gamma = 3$ and $b_\gamma = 6$ (here falling below the base case value), but for $b_\gamma = 9$ the core size *increases*. It seems that, in this case, the extent of lipid removal from the plaque is so substantial that recruitment is reduced and too few macrophages remain in the plaque to resolve the necrotic core. This explanation is corroborated by the steady state $m\left( a,t\right)$ distribution in Fig. 10d (green line). Compared to the corresponding result for the unscaled case (Fig. 10c), we see large reductions in both the proportion of cells with very large lipid loads (see inset) and the proportion of cells with very small lipid loads ($a \approx 1$). The cases for $b_\gamma = 3$ and $b_\gamma = 6$ (red and blue lines, respectively) show similarly large reductions in *m* at the top end of their distributions, but at the lower end both distributions increase.

### Lipid-Dependent Simulations with Proliferation

In this section, we introduce macrophage proliferation into the system by setting $\rho = 0.5$. First we assume that proliferation is independent of lipid load. We briefly revisit the scenarios from Sect. 3.2 to study the manner in which proliferation (with its tendency to reduce average lipid loads) interacts with lipid-dependence in the other cell behaviours. We then revert to lipid-independent apoptosis and emigration, and conclude the results by investigating the impact of lipid-dependence in proliferation itself.

#### Apoptosis Only or Emigration Only

We set $g_\rho \left( a\right) = 1$, and select one lipid-dependent case from each of Sects. 3.2.1 and 3.2.2. These are $g_\beta = g_\beta ^s$ with $a_\beta = 12$, $\delta _\beta = 3$ and $g_\gamma = g_\gamma ^s$ with $a_\gamma = 18$, respectively. As before, we allow lipid-dependence in only one cell behaviour at a time, and we investigate each lipid-dependent case using both scaled and unscaled functions. (Note that $g_\beta$ and $g_\gamma$ generally require new scaling values to account for the impact of proliferation; see Table 2). Here, we shall primarily focus on the scaled cases since preserving the net steady state apoptosis or emigration rate facilitates comparison against the earlier scaled cases without proliferation. The unscaled cases do, however, provide some interest and we comment on these below.

For the unscaled lipid-dependent apoptosis case, Table 2 shows that the inclusion of proliferation acts to reduce the net steady state apoptosis rate $G_\beta$ by approximately 8% from 1.576 to 1.445. This reflects the fact that proliferation acts to reduce individual cell lipid loads, thereby reducing the likelihood of apoptosis (i.e. daughter cell apoptosis rates $g_\beta ^s\left( a\right)$ are always less than parent cell apoptosis rates $g_\beta ^s\left( 2a-1\right)$). Note, however, that a tangible reduction in the daughter cell apoptosis rate requires $g_\beta ^s\left( a\right) \ll g_\beta ^s\left( 2a-1\right)$, and this occurs only for daughter cells with *a* sufficiently small. The unscaled lipid-dependent emigration case produces the rather curious result that proliferation actually *reduces* the net steady state emigration rate ($G_\gamma \left( \infty \right)$ drops from 0.7812 to 0.7650; see Table 2). This is counter-intuitive because the associated reduction of lipid loads would be expected to *increase* the likelihood of emigration (i.e. $g_\gamma ^s\left( a\right) > g_\gamma ^s\left( 2a-1\right)$ for all *a*). This quirk appears to arise due to the proliferation of cells with very large lipid loads. Proliferation of such cells fails to significantly elevate daughter cell emigration rates because $g_\gamma ^s\left( a\right)$ remains similar to $g_\gamma ^s\left( 2a-1\right)$ for large *a*. Thus, lipid loaded cells with very low emigration rates perpetuate in the system and skew the $G_\gamma$ value upwards. Despite this unexpected increase in $G_\gamma$, proliferation does, as expected, reduce the average lipid per cell at steady state.

**Fig. 13:**
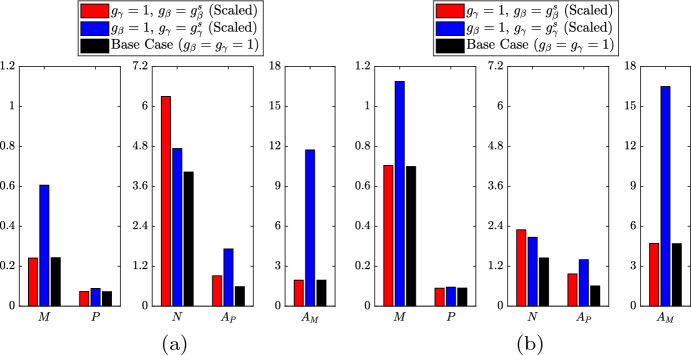
Steady state solutions of the ODE variables $M\left( t\right)$, $P\left( t\right)$, $N\left( t\right)$, $A_P\left( t\right)$ and $A_M\left( t\right)$ for lipid-independent ($g_\beta = g_\gamma = 1$; black bars) and scaled lipid-dependent cases **a** without proliferation ($\rho = 0$) and **b** with proliferation ($\rho = 0.5$). Red bars show results with lipid-dependent apoptosis ($g_\beta = g_\beta ^s$, $g_\gamma = 1$) using parameter values $n_\beta = 2$, $\delta _\beta = 3$ and $a_\beta = 12$. Blue bars show results with lipid-dependent emigration ($g_\gamma = g_\gamma ^s$, $g_\beta = 1$) using parameter values $n_\gamma = 1.5$, $\delta _\gamma = 0.1$ and $a_\gamma = 18$ (Color figure online)

**Fig. 14:**
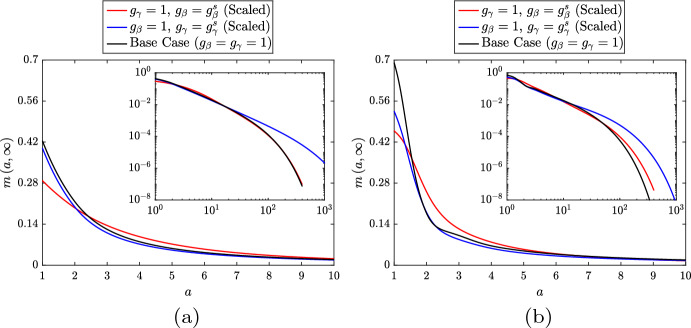
Steady state $m\left( a,t\right)$ distributions for lipid-independent ($g_\beta = g_\gamma = 1$; black bars) and scaled lipid-dependent cases **a** without proliferation ($\rho = 0$) and **b** with proliferation ($\rho = 0.5$). Red lines show results with lipid-dependent apoptosis ($g_\beta = g_\beta ^s$, $g_\gamma = 1$) using parameter values $n_\beta = 2$, $\delta _\beta = 3$ and $a_\beta = 12$. Blue lines show results with lipid-dependent emigration ($g_\gamma = g_\gamma ^s$, $g_\beta = 1$) using parameter values $n_\gamma = 1.5$, $\delta _\gamma = 0.1$ and $a_\gamma = 18$. Primary plots show the results on the interval $a \in \left[ 1,10\right]$ and inset log–log plots show the results on the entire *a* domain (Color figure online)

Returning to the scaled lipid-dependent functions, Fig. 13 presents the steady state ODE solutions for the lipid-dependent and base case simulations both in the absence (Fig. 13a) and presence (Fig. 13b) of macrophage proliferation. Although the two solution sets are quantitatively different, the lipid-dependent results display the same qualitative trends regardless of the particular proliferation rate. Lipid-dependent apoptosis increases *N* and $A_P$ (*M*, *P* and $A_M$ remain fixed), while lipid-dependent emigration increases the solutions of all five ODE variables. Figure 14 shows the corresponding steady-state $m\left( a,t\right)$ distributions for the cases without proliferation (Fig. 14a) and with proliferation (Fig. 14b). As proliferation adds a second non-local effect into the model, the precise features of the plots in Fig. 14b are difficult to interpret. However, as expected based on Eq. (35), proliferation tends to shift the $m\left( a,t\right)$ distributions towards lower accumulated lipid loads. For lipid-dependent apoptosis, proliferation produces a relatively large increase in the proportion of cells with small lipid loads. However, for large *a*, the impact of proliferation seems relatively minimal. This likely reflects the substantial increase in the apoptosis rate with increasing *a*, which effectively acts to suppress proliferation events.

#### Proliferation Only

**Fig. 15:**
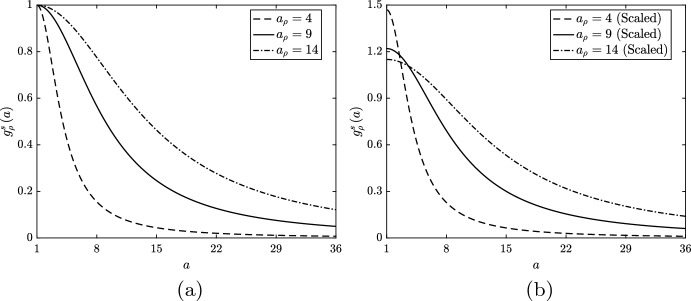
**a** Unscaled and **b** scaled rate modulating functions for macrophage proliferation $g_\rho \left( a\right) = g_\rho ^s\left( a\right)$. Plots correspond to Eq. (32) with parameter values $n_\rho = 2$, $\delta _\rho = 0$ and $a_\rho = 4$ (dashed lines), $a_\rho = 9$ (solid lines) or $a_\rho = 14$ (dot-dashed lines). Scaling values for the plots in **b** are 1.47, 1.22 and 1.15, respectively

Finally, we investigate the role of lipid-dependent macrophage proliferation. In this section, we set $g_\rho \left( a\right) = g_\rho ^s\left( a\right)$ and fix $g_\beta \left( a\right) = g_\gamma \left( a\right) = 1$. Once again, we consider three different forms for the unscaled lipid dependent functions (Fig. 15a). These functions have $n_\rho = 2$, $\delta _\rho = 0$ and $a_\rho = 4$, 9 or 14. By varying $a_\rho$, we study the effect of different rates of decline in the macrophage proliferation rate with increased lipid loading (Kim et al. 2018).

**Fig. 16:**
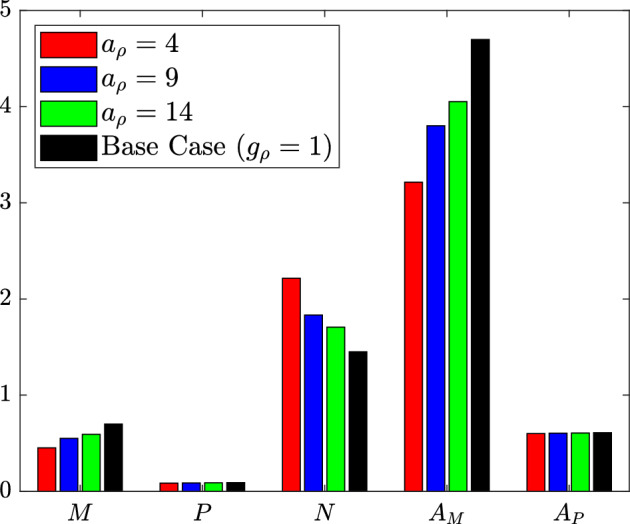
Steady state solutions of the ODE variables $M\left( t\right)$, $P\left( t\right)$, $N\left( t\right)$, $A_P\left( t\right)$ and $A_M\left( t\right)$ for the lipid-independent base case ($g_\rho = 1$; black bars) and for three cases with unscaled lipid-dependent proliferation ($g_\rho = g_\rho ^s$). Lipid-dependent cases have parameter values $n_\rho = 2$, $\delta _\rho = 0$ and $a_\rho = 4$ (red bars), $a_\rho = 9$ (blue bars) or $a_\rho = 14$ (green bars) (Color figure online)

The steady state ODE solutions generated using these functions are presented in Fig. 16. Here, we observe marginal decreases in *P* and $A_P$, marked decreases in *M* and $A_M$, and a marked increase in *N* with decreasing $a_\rho$. These variations in the steady state solutions across the lipid-dependent cases are not explicitly related to the particular form of $g_\rho$, but simply reflect the associated changes in the net proliferation rates $\rho G_\rho \left( \infty \right)$. The case with $a_\rho = 4$, for example, has net steady state proliferation rate $\rho G_\rho \approx \left( 0.5\right) \left( 0.6377\right) \approx 0.319$. A lipid-independent simulation ($G_\rho = 1$) with proliferation rate $\rho = 0.319$ would therefore give almost identical steady state ODE solutions, albeit with different temporal dynamics and different steady state PDE solutions.

It is interesting to observe that the $G_\rho$ values in these lipid-dependent simulations remain fairly close to 1, even when $g_\rho ^s$ declines rapidly with *a*. This suggests that the model is relatively insensitive to the value of the parameter $a_\rho$, provided it is not too close to 1. This makes intuitive sense because proliferation generally acts to keep cell lipid loads close to $a=1$, and, provided $a_\rho$ is not too small, the lipid-dependent proliferation rate remains high throughout this region (i.e. $g_\rho ^s \approx 1$). Contrastingly, while lipid-independent proliferation may allow some cells to acquire small lipid loads from large lipid loads through multiple proliferation events, this phenomenon will be suppressed in the lipid-dependent cases due to the decline in $g_\rho ^s$ for large *a*.

**Fig. 17:**
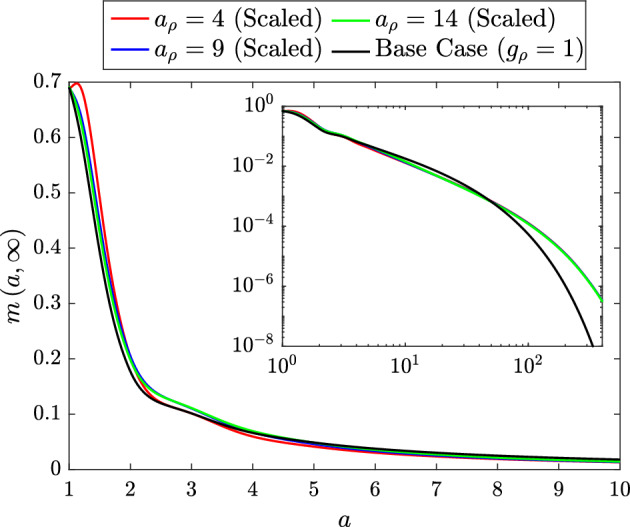
Steady state $m\left( a,t\right)$ distributions for the lipid-independent base case ($g_\rho = 1$; black lines) and for three cases with scaled lipid-dependent proliferation ($g_\rho = g_\rho ^s$). Lipid-dependent cases have parameter values $n_\rho = 2$, $\delta _\rho = 0$ and $a_\rho = 4$ (red lines), $a_\rho = 9$ (blue lines) or $a_\rho = 14$ (green lines). The primary plot shows the results on the interval $a \in \left[ 1,10\right]$ and the inset log–log plot shows the results on the entire *a* domain. All four distributions coincide at $a = 1$ because the values of the ODE variables in the boundary condition (30) are almost identical in each case (Color figure online)

Consistent with their definition, simulations using the scaled $g_\rho ^s$ (Fig. 15b) lead to net proliferation rates approximately equal to $\rho$ at steady state. Accordingly, each of these simulations produces steady state ODE results that are indistinguishable from the base case. We therefore omit these ODE results and focus instead on the corresponding steady state $m\left( a,t\right)$ distributions (Fig. 17). We also omit the $m\left( a,t\right)$ distributions generated using the unscaled $g_\rho ^s$. These differ slightly from the scaled $g_\rho ^s$ results for small *a*, but the distributions become increasingly similar as *a* increases. Figure 17 shows that there are few discernible differences between the base case steady state $m\left( a,t\right)$ distribution and the distributions generated using the scaled $g_\rho ^s$. One exception is that the lipid-dependent cases all have a larger proportion of cells with lipid load $a>50$. This is because these cells have substantially reduced proliferation rates and cannot readily divide to reduce their lipid load. In the case with $a_\rho = 4$, there is a subtle change in the form of the distribution with the emergence of a small peak near $a=1$. This is likely due to the locally elevated proliferation rate, which alters the relative balance between proliferation and efferocytosis (see Fig. 7 in Chambers et al. (2022)). Overall, we conclude from the results in this section that the outcome of plaque formation is probably more sensitive to the net population-level proliferation rate than to the particular lipid-dependent distribution of proliferation rates across the population.

## Discussion

Lipid consumption is known to alter macrophage behaviour but the implications for atherosclerotic plaque formation are not well understood. In this paper, we establish a novel modelling framework to study these implications. We develop a structured population model of plaque macrophage lipid accumulation in which macrophages are classified by their internalised lipid load *a* and behave in a lipid-dependent manner. This work builds upon the partial integro-differential equation models recently developed in Ford et al. (2019a) and Chambers et al. (2022). As in these earlier works, we model how live macrophage behaviour feeds into apoptotic macrophage population dynamics and necrotic core formation through mechanisms such as efferocytosis, post-apoptotic necrosis and necrotic core consumption. A key difference in the current model is that the lipid-averaged ODE subsystem cannot be easily decoupled from the governing PDEs.

We focus our investigation on how lipid-dependent macrophage apoptosis, emigration and proliferation can influence plaque progression. This work is based on results that show dysfunctionality in macrophages that have large ingested lipid loads (Tabas 2002; Moore et al. 2013; Yin and Heit 2021). Consequently, we assume that increasing lipid loads cause increased rates of apoptosis and reduced rates of both emigration and proliferation. We do not consider lipid-induced dysfunction in macrophage phagocytic or efferocytic capacity. Thus, heavily lipid-loaded macrophages in our simulations consume extracellular lipid and engulf apoptotic bodies at the same rate as cells with small ingested lipid loads. This is a limitation of the current study that we will address in future by including lipid-dependent phagocytosis and efferocytosis terms in the model. We anticipate that the inclusion of these terms will have dual benefit. Not only will the model become more realistic, but lipid consumption rates that decrease with increasing lipid load will reduce the domain sizes required for accurate numerical solutions. Model simulations will therefore be less computationally demanding and numerical techniques such as the non-uniform gridding strategy implemented here may no longer be required.

Lipid-dependent macrophage behaviour is encoded in the model through the dimensionless functions $g_\beta \left( a\right)$, $g_\gamma \left( a\right)$ and $g_\rho \left( a\right)$, which modulate the reference rates of apoptosis, emigration and proliferation, respectively. These functions typically take a monotonic form, but for emigration we also consider a non-monotonic lipid-dependence. In the absence of appropriate data to parameterise these functions, we simulate a range of scenarios with different rates of approach to their (finite) limiting values. This is a reasonable strategy as the lipid-dependence of each behaviour *in vivo* is likely to be controlled by several different factors including the activation or disruption of unique signalling pathways (Feng et al. 2003; van Gils et al. 2012; Robbins et al. 2013). Thus, the rate of each individual macrophage behaviour may be altered in different ways, at different times and at different internalised lipid loads. In our analysis, we have made the simplifying assumption that only one of apoptosis, emigration or proliferation may be lipid-dependent at any one time. While this may be unrealistic in practice, the benefit of this assumption is that we can study the influence of each lipid-dependent behaviour on plaque progression. Of course, simulations with more than one lipid-dependent behaviour can be easily performed within our model framework. However, the increased difficulty in interpretation of such results limits the insight that can be gained.

For each scenario that we model in this paper, we fix the lipid-independent parameter values and perform several simulations using both unscaled and scaled functions $g_\diamond \left( a\right)$. Unscaled simulations use the exact functional forms (32) or (33) with an appropriate set of lipid-dependent parameter values. Each corresponding scaled simulation uses the same lipid-dependent parameter values, but the function $g_\diamond$ is multiplicatively scaled such that the net rate of the behaviour of interest matches the reference rate at steady state (mathematically, this is expressed as $G_\diamond \left( \infty \right) = \int _1^\infty g_\diamond \left( a\right) m\left( a,\infty \right) \, da = 1$ where $g_\diamond \left( a\right) \ne 1$). Each approach has unique advantages and disadvantages. Unscaled simulations are relevant for understanding the impact of variability in lipid-dependent macrophage behaviour and/or the response of the model system to interventions that alter the lipid-dependence. However, as the net steady state rate of the behaviour of interest is not conserved from case to case, it is difficult to interpret the impact of the lipid-dependence itself. By correcting for this, scaled simulations provide a more consistent and insightful means of comparison across simulated scenarios. However, the scaling value required in a given case is not known *a priori* and must be determined by a process of estimation and refinement through repeated simulation. As such, the scaling process itself can be computationally demanding.

Our results and analysis indicate some interesting population level differences in the relative influence of lipid-dependent apoptosis, emigration and proliferation on plaque progression. For unscaled lipid-dependent proliferation simulations with net steady state proliferation rate $\rho G_\rho \left( \infty \right)$, we find that the steady state ODE solutions *M*, *P*, $A_M$, $A_P$ and *N* can always be matched by an equivalent lipid-independent simulation with the constant proliferation rate $\rho G_\rho \left( \infty \right)$. Consequently, for all scaled lipid-dependent apoptosis simulations ($G_\rho \left( \infty \right) \approx 1$), we obtain essentially identical steady state ODE results. This phenomenon is unique to lipid-dependent proliferation and equivalent outcomes are not seen for lipid-dependent apoptosis or lipid-dependent emigration simulations. Mathematically, this reflects the presence in the model equations of the terms $G_{\beta a}$ and $G_{\gamma a}$. These terms appear because the net rate of lipid removal from the live cell population by lipid-dependent apoptosis or lipid-dependent emigration depends on the distribution of lipid across the population. There is no equivalent $G_{\rho a}$ term in the model equations because the quantity of lipid added to the system by a proliferation event ($a_0$ in dimensional terms) is always fixed and does not depend on the internalised lipid content of the parent cell. Thus, compared to lipid-dependent proliferation, the model equations for lipid-dependent apoptosis or lipid-dependent emigration have additional nonlinear terms that increase the complexity of the underlying dynamics. This is emphasised by Eq. (35), where we see that lipid-dependent apoptosis and lipid-dependent emigration can dynamically alter ${\bar{A}}_M\left( t\right)$ in a way that is not observed at all in lipid-independent cases.

In our unscaled lipid-dependent apoptosis simulations, we observe the emergence of decaying oscillations in the ODE variables when the increase in $g_\beta \left( a\right)$ is sufficiently large in both magnitude and steepness. Oscillatory solutions of this type appear to be unique to the lipid-dependent model as they have not been observed in earlier lipid-independent approaches (Ford et al. 2019a; Chambers et al. 2022). Two factors that appear to be necessary to initiate these oscillations are: (1) macrophages ingest (primarily necrotic) lipid at such a rate that $A_M$ grows irrespective of a concurrent increase in the net apoptosis rate; and (2) this increase in $A_M$ stimulates an increase in macrophage recruitment. The latter requirement may only be satisfied for certain values of the dimensionless parameter $\kappa$. We use $\kappa = 5$ in our simulations. However, for a smaller $\kappa$ value, the recruitment rate would probably be less sensitive to changes in $A_M$ and it is possible that oscillations may not emerge at all.

The observation that oscillatory solutions require a sufficiently rapid rate of lipid ingestion relative to apoptosis raises an interesting question about what might happen if $g_\beta$ does not saturate and instead approaches infinity at some finite *a*; that is, if there exists a certain lipid load above which macrophages become unviable. It is not clear whether oscillations could occur in this case because $A_M$ may be unable to grow due to (potentially) very rapid lipid-induced apoptosis. Non-saturating lipid-dependent death rates may be generally interesting to investigate in this model because, together with lipid ingestion rates that decrease with *a*, they offer another means to reduce (and, indeed, cap) the length of the *a* domain.

Oscillatory solutions have also been observed in a plaque formation model by Bulelzai et al. (2014). While it is possible that there may be underlying similarity in the mathematical structure of the models, the biological mechanisms that produce these oscillations in the respective models are quite distinct. These independent observations naturally raise the question as to whether such oscillations are biologically realistic. To our knowledge, oscillatory behaviour in real plaques has never been reported experimentally. However, most plaque data is collected *ex vivo* and *in vivo* plaques are typically not observed with sufficient resolution or sufficiently often for such an observation to be made. The oscillations reported here reflect a particular set of modelling assumptions, and, in practice, there may be other factors (e.g., spatial effects, other lipid-dependent behaviours) that act to modify the dynamics. However, the dynamic feedback inherent in monocyte/macrophage recruitment, as well as the multiple timescales inherent in plaque progression, do support the plausibility of oscillatory dynamics in real plaques.

For scaled lipid-dependent apoptosis, we find the surprising result that the steady state *M*, *P* and $A_M$ values remain unchanged across all simulated cases. Our analysis shows that this happens because, at steady state in each simulation, the net rate of lipid loss from live cells due to apoptosis ($G_{\beta a}$) is exactly balanced by the rate of lipid gain via efferocytosis and necrotic lipid consumption ($\eta A_P + \theta N$). The balance between these terms fixes the steady state $A_M$ value in each simulation, and fixed steady state *M* and *P* follow because the recruitment rate, net apoptosis rate and all other relevant rates remain unchanged. Of course, while the relationship between $G_{\beta a}$ and $\eta A_P + \theta N$ causes steady state *M*, *P* and $A_M$ to remain fixed, the very same relationship causes steady state $A_P$ and *N* to vary. The steady state values of $G_{\beta a}$, $A_P$ and *N* all increase with increasing magnitude and steepness of change in scaled $g_\beta ^s$. These results suggest that plaques whose cells have increased susceptibility to the cytotoxic effects of lipid accumulation can have larger necrotic cores at steady state even if the net macrophage apoptosis rate is held fixed. Based on the ODE for total system lipid $L\left( t\right)$, we further propose that this result reflects excess lipid accumulation in the plaque over time, primarily due to a sustained period of reduced lipid removal by emigration.

Our results for lipid-dependent emigration are arguably the most difficult to analyse and interpret because, even in the case of scaled $g_\gamma \left( a\right)$, none of the steady state ODE solutions remain fixed across our simulated cases. This reflects the influence of the term $G_{\gamma a}$ on the dynamics of $A_M$, which then affects the other ODE variables via the macrophage recruitment rate. Although not considered here, our results suggest that it may be worthwhile to perform a complementary set of lipid-dependent simulations where the functions $g_\gamma$ are scaled such that, at steady state, the quantity $G_{\gamma a}$ matches its base case value; that is, scale the $g_\gamma$ to match the rate of lipid removal by macrophage emigration at steady state rather than the rate of macrophage emigration itself. While this approach is unlikely to keep any of the individual steady state ODE solutions at their base case values, it may give additional insight into the intricacies of the simulated outcomes.

In our simulations with unscaled monotonic lipid-dependent emigration, we find that the steady state values of the ODE variables all increase with increasing rate of decline of $g_\gamma$ (decreasing $a_\gamma$). This is unsurprising because, as $a_\gamma$ decreases, the net emigration rate also decreases and the system therefore retains more cells and more lipids. In simulations with scaled $g_\gamma$, we observe the exact same trend in the steady state ODE values. This shows that the reduced net emigration rate in the unscaled cases is only partially responsible for the observed outcomes and, in fact, it is the form of the lipid-dependence that contributes much of the increase in the steady state ODE values relative to the base case. This observation reflects the fact that any cell that fails to emigrate for *a* small becomes increasingly unlikely to emigrate at all. Cells can therefore persist in the system, acquiring lipid and eventually undergoing apoptosis. This leads to large ingested lipid quantities in both the live and apoptotic cell populations, which ultimately feed into enlarged necrotic cores. Our results show that the detrimental effects of monotonically decreasing lipid-dependent emigration can be substantial. Scaled simulations predict up to a 40% increase in necrotic core size despite a 2- to 3-fold increase in the net rate of necrotic core consumption.

As well as monotonic lipid-dependent emigration, we consider non-monotonic $g_\gamma$ where macrophages with moderate lipid loads have the highest emigration rates. These $g_\gamma$ impose reduced rates of emigration for macrophages with very small lipid loads on the basis that such cells (in the absence of proliferation) seem unlikely to exit the plaque (Llodrá et al. 2004). Our results show that non-monotonic lipid-dependent emigration can be beneficial relative to the monotonic lipid-dependence. As cells are more likely to emigrate with a relatively large lipid load, this not only removes more lipid from the system but it also (in scaled cases at least) reduces the likelihood that cells acquire large lipid loads before dying. Our simulations with parameter value $b_\gamma = 9$ are particularly interesting. In the unscaled case, we see a reduction in steady state *N* relative to the base case. This is particularly remarkable because: (1) the steady state $G_\gamma$ is only half of the base case value; and (2) the emigration rate of cells with very large *a* is up to 10 times smaller than in the base case. In the equivalent scaled case, however, we see a larger steady state *N* relative to the base case. This is because extensive lipid removal from the system by emigration inhibits the immune response. Taken together, these results suggest that the presence of at least some heavily lipid-loaded (and highly inflammatory) macrophages in the plaque may be beneficial for stimulating the recruitment that is required for necrotic core consumption. Observations such as this may be useful in the interpretation of results from experimental studies on plaque resolution and regression (Rahman and Fisher 2018).

Our lipid-dependent emigration results provide new insight into how the distribution of emigration events can influence the extent of lipid removal from the plaque and the overall plaque progression. These results, however, should be interpreted with caution. We assume that the likelihood of macrophage emigration depends only on lipid load, whereas, in practice, this relationship would be time- and space-dependent due to factors such as plaque size and the strength of emigratory signals relative to other migratory cues (e.g. “find me” signals from apoptotic cells (Kojima et al. 2017)). The inclusion of spatial effects in future models will allow a better understanding of how macrophage emigration events may be distributed with respect to internalised lipid loads, both over time and at steady state.

In the current work, we perform two sets of simulations that include macrophage proliferation. We first investigate how lipid-independent proliferation interacts with lipid-dependence in the other kinetic terms, and we then investigate the impact of a monotonically decreasing lipid-dependent proliferation rate. When lipid-independent proliferation is combined with scaled lipid-dependent apoptosis or emigration, we observe, as expected, a general reduction in necrotic core size and average cell lipid loads. Overall, however, the qualitative trends in the steady state ODE solutions remain the same as in the proliferation-free cases. When $g_\beta$ is unscaled, we make the interesting observation that proliferation can improve the outcome of plaque formation by reducing the net apoptosis rate. Although the observed reduction at steady state is relatively small (around 8%), the lipid-dependent apoptosis rates range from 2–6 times larger than the proliferation rate (and, as observed in proliferation-free cases, lipid-dependent apoptosis tends to drive cells to higher *a* by increasing lipid consumption). For simulations with lipid-dependent proliferation, we identify two main findings. First, that the steady state ODE solutions depend only on the net steady state proliferation rate $\rho G_\rho$, and, second, that the steady state distribution of lipid across the live cell population is relatively insensitive to the form of $g_\rho$ (provided steady state $G_\rho$ is not too far from 1).

Although we consider a monotonically decreasing form for $g_\rho$, there exists experimental evidence that the rate of lipid-dependent macrophage proliferation may peak at an intermediate lipid content (Xu et al. 2015). This form of lipid-dependent proliferation can be easily included in the model by allowing $g_\rho$ to take the non-monotonic form defined in Eq. (33). An interesting implication of this non-monotonic lipid-dependence is that, unlike monotonically decreasing lipid-dependent proliferation, it can naturally explain the observation that the contribution of plaque macrophage proliferation increases over time (Lhoták et al. (2016); i.e. as plaque-resident macrophages gradually accumulate lipid, the net population proliferation rate would grow). Of course, many other factors may contribute to this phenomenon, including the presence of a fibrous cap in mature plaques that may inhibit the rate of monocyte recruitment from the bloodstream. We have performed preliminary model investigations using non-monotonic lipid-dependent proliferation, but we omit the results here because we find that they are not substantially different to those with monotonic lipid-dependence. However, in simulations that consider more than one lipid-dependent behaviour at a time (e.g. lipid-dependent proliferation and lipid-dependent apoptosis), we envisage that the particular form of lipid-dependent proliferation may have a more significant impact on the outcome of plaque progression. For example, a lipid-dependent macrophage proliferation rate that peaks close to the lipid load at which the lipid-dependent apoptosis rate begins to rise would potentially optimise the protective effect of proliferation against the otherwise detrimental effects of lipid cytotoxicity.

Before concluding, we emphasise that the aim of this study is to use mathematical modelling to identify ways in which lipid-dependent macrophage behaviour may modify atherosclerotic plaque fate and dynamics. We also wish to understand, from a mathematical perspective, the mechanisms that underlie these modifications. By design, we consider the implications of several different lipid-dependent macrophage behaviours in isolation. This approach provides an ideal way to achieve the stated aims. However, as this model setup is somewhat artificial, we remain naturally cautious when interpreting our findings with respect to *in vivo* plaque formation. Real plaques are likely to have multiple different lipid-dependent macrophage behaviours at play at any given time and, hence, direct extrapolation of our findings to existing *in vivo* observations is challenging. The real significance of this work is that it demonstrates that the effects of lipid-dependent macrophage behaviour on plaque formation can be substantial, and therefore highlights that more detailed experimental measurements (including, for example, macrophage internalised lipid distributions) will almost certainly be required to fully comprehend the mechanisms of *in vivo* plaque progression.

## Conclusions

This paper extends the work of Ford et al. (2019a) and Chambers et al. (2022) by developing a lipid-structured model of atherosclerotic plaque macrophages in which the rates of macrophage apoptosis, emigration and proliferation are modulated by the internalised lipid load. We model these lipid-dependent behaviours using dimensionless modulating functions whose features align qualitatively with a range of experimental observations. Our results indicate, particularly for apoptosis and emigration, that variations in macrophage behaviour across lipid loads can substantially alter plaque fate relative to cases without lipid-dependence. We find that these changes to plaque fate are difficult to predict because the lipid-dependent behaviours introduce subtle, nonlinear effects that feed back into the model through phagocytosis, efferocytosis and recruitment. This work provides new insight into how macrophage lipid accumulation can shape atherosclerotic plaque progression and highlights the importance of mathematical modelling as a tool in the scientific study of atherosclerotic plaque macrophages.

## Data Availability

Data sharing not applicable to this article as no datasets were generated or analysed during the current study.

## Appendix A: Dimensionless Equations

With tildes dropped for notational convenience, the dimensionless model equations given by the scalings (19) and (20) are:

23 $$\begin{aligned} \begin{aligned}&\frac{\partial m\left( a,t\right) }{\partial t} + \bigg [\frac{\lambda }{M\left( t\right) } + \theta N\left( t\right) \bigg ] \frac{\partial m\left( a,t\right) }{\partial a} \\&\quad = \eta P\left( t\right) \bigg [\int _{1}^{a-1} m(a',t) p(a-a',t) \, da' - m\left( a,t\right) \bigg ] \\&\qquad + \bigg [\Big (G_\beta \left( t\right) - g_\beta \left( a\right) \Big ) + \gamma \, \Big (G_\gamma \left( t\right) - g_\gamma \left( a\right) \Big ) \bigg ] m\left( a,t\right) \\&\qquad + 4 \rho g_\rho \left( 2a-1\right) m(2a-1,t) - \Big (G_\rho \left( t\right) + g_\rho \left( a\right) \Big )\rho m\left( a,t\right) \\&\qquad - \bigg [\frac{1}{M\left( t\right) } \, \frac{A_M\left( t\right) - M\left( t\right) }{\kappa + A_M\left( t\right) - M\left( t\right) }\bigg ] m\left( a,t\right) , \end{aligned} \end{aligned}$$

24 $$\begin{aligned} \frac{\partial p\left( a,t\right) }{\partial t} = \frac{M\left( t\right) }{P\left( t\right) } \, \Big [g_\beta \left( a\right) m\left( a,t\right) - G_\beta \left( t\right) p\left( a,t\right) \Big ], \end{aligned}$$

25 $$\begin{aligned} \frac{dN\left( t\right) }{dt} = \nu A_P\left( t\right) - \theta M\left( t\right) N\left( t\right) , \end{aligned}$$

26 $$\begin{aligned} \frac{dM\left( t\right) }{dt} = \frac{A_M\left( t\right) - M\left( t\right) }{\kappa + A_M\left( t\right) - M\left( t\right) } - \Big [G_\beta \left( t\right) + \gamma G_\gamma \left( t\right) - \rho G_\rho \left( t\right) \Big ] M\left( t\right) , \end{aligned}$$

27 $$\begin{aligned} \begin{aligned} \frac{dA_M\left( t\right) }{dt} = \frac{A_M\left( t\right) - M\left( t\right) }{\kappa + A_M\left( t\right) - M\left( t\right) } + \lambda + \theta M\left( t\right) N\left( t\right) \\ + \eta M\left( t\right) A_P\left( t\right) - \Big [G_{\beta a}\left( t\right) + \gamma G_{\gamma a}\left( t\right) - \rho G_\rho \left( t\right) \Big ] M\left( t\right) , \end{aligned} \end{aligned}$$

28 $$\begin{aligned} \frac{dP\left( t\right) }{dt} = G_\beta \left( t\right) M\left( t\right) - \Big [\nu + \eta M\left( t\right) \Big ] P\left( t\right) , \end{aligned}$$

29 $$\begin{aligned} \frac{dA_P\left( t\right) }{dt} = G_{\beta a}\left( t\right) M\left( t\right) - \Big [\nu + \eta M\left( t\right) \Big ] A_P\left( t\right) . \end{aligned}$$

The boundary condition is:

30 $$\begin{aligned} \bigg [\frac{\lambda }{M\left( t\right) } + \theta N\left( t\right) \bigg ] m\left( 1,t\right) = \frac{1}{M\left( t\right) } \, \frac{A_M\left( t\right) - M\left( t\right) }{\kappa + A_M\left( t\right) - M\left( t\right) }, \end{aligned}$$

and the initial conditions are:

31 $$\begin{aligned} \begin{aligned} m_0\left( a\right)&= p_0\left( a\right) = \frac{2}{a_\sigma \sqrt{2\pi }} \, \exp \left( - \, \frac{\left( a - 1\right) ^2}{2 {a_\sigma }^2}\right) , \\ N_0&= 0, \\ M_0&= 2P_0 = \frac{\kappa \lambda \sqrt{2 \pi }}{a_\sigma \Big [a_\sigma \sqrt{2 \pi } - 2 \lambda \Big ]}, \\ A_{M0}&= 2A_{P0} = M_0\left( 1 + \frac{2}{\sqrt{2\pi }} \, a_\sigma \right) . \end{aligned} \end{aligned}$$

We set $a_\sigma = 0.5$ for all simulations in this paper.

The functions that control lipid-dependent cell behaviour are:

32 $$\begin{aligned} g_\diamond ^s\left( a\right)= & {} \frac{\left( a_\diamond - 1\right) ^{n_\diamond } + \delta _\diamond \left( a - 1\right) ^{n_\diamond }}{\left( a_\diamond - 1\right) ^{n_\diamond } + \left( a - 1\right) ^{n_\diamond }}, \end{aligned}$$

33

The integral terms in (23)–(29) are $G_\diamond \left( t\right) = \int _{1}^{\infty } g_\diamond \left( a\right) m\left( a,t\right) \, da$ and $G_{\diamond a}\left( t\right) = \int _{1}^{\infty } g_\diamond \left( a\right) a m\left( a,t\right) \, da$. In the case of lipid independence (i.e., $g_\diamond \left( a\right) \equiv 1$), these integrals evaluate to $G_\diamond \left( t\right) = 1$ and $G_{\diamond a}\left( t\right) = \frac{A_M\left( t\right) }{M\left( t\right) }$.

## Appendix B: Total System Lipid and Average Lipid Per Cell

The ODE system (25)–(29) can be used to derive additional ODEs for the total amount of lipid in the system $L\left( t\right)$, the average lipid content per live cell ${{\bar{A}}}_M\left( t\right)$, and the average lipid content per apoptotic cell ${{\bar{A}}}_P\left( t\right)$. In what follows, we make the definition $F\left( t\right) = \frac{A_M\left( t\right) - M\left( t\right) }{\kappa + A_M\left( t\right) - M\left( t\right) }$, where $F\left( t\right)$ is proportional to the dimensionless macrophage recruitment rate.

For $L\left( t\right)$, we have:

34 $$\begin{aligned} \frac{dL\left( t\right) }{dt} = F\left( t\right) + \lambda + \rho G_\rho \left( t\right) M\left( t\right) - \gamma G_{\gamma a}\left( t\right) M\left( t\right) . \end{aligned}$$

The mechanisms that add lipid to the system are monocyte recruitment, LDL consumption, and local macrophage proliferation (first, second and third terms on the right-hand side). Contrastingly, the sole mechanism of lipid removal from the system is macrophage emigration (final term on the right-hand side). The time evolution of $L\left( t\right)$ is driven entirely by the behaviour of live macrophages, while explicit lipid-dependent effects appear only in the proliferation and emigration terms.

For ${{\bar{A}}}_M\left( t\right)$, we have:

35 $$\begin{aligned} \begin{aligned} \frac{d{{\bar{A}}}_M\left( t\right) }{dt}&= \frac{\lambda }{M\left( t\right) } + \theta N\left( t\right) + \eta A_P\left( t\right) \\&\quad - \frac{F\left( t\right) }{M\left( t\right) } \Big [{{\bar{A}}}_M\left( t\right) - 1\,\Big ] - \rho G_\rho \left( t\right) \Big [{{\bar{A}}}_M\left( t\right) - 1\,\Big ] \\&\quad + \Big [G_\beta \left( t\right) {{\bar{A}}}_M\left( t\right) - G_{\beta a}\left( t\right) \Big ] + \gamma \Big [G_\gamma \left( t\right) {{\bar{A}}}_M\left( t\right) - G_{\gamma a}\left( t\right) \Big ]. \end{aligned} \end{aligned}$$

The first three terms on the right-hand side show that LDL consumption, necrotic lipid consumption and efferocytosis all act to increase ${{\bar{A}}}_M\left( t\right)$. On the other hand, monocyte recruitment and macrophage proliferation (terms four and five) always act to reduce ${{\bar{A}}}_M\left( t\right)$ (note that ${{\bar{A}}}_M\left( t\right) > 1$ in all but the extreme case where $m\left( a,t\right)$ is a delta distribution). The final two terms, which relate to apoptosis and emigration, can act to either increase or decrease ${{\bar{A}}}_M\left( t\right)$ depending on their signs. Letting $\diamond$ denote $\beta$ or $\gamma$, we see that these terms act to increase ${{\bar{A}}}_M\left( t\right)$ when $\frac{G_{\diamond a}\left( t\right) }{G_\diamond \left( t\right) } < {{\bar{A}}}_M\left( t\right)$ and act to decrease ${{\bar{A}}}_M\left( t\right)$ when $\frac{G_{\diamond a}\left( t\right) }{G_\diamond \left( t\right) } > {{\bar{A}}}_M\left( t\right)$. The ratio $\frac{G_{\diamond a}\left( t\right) }{G_\diamond \left( t\right) }$ can be interpreted as the average lipid load of cells leaving the live cell population via apoptosis or emigration at time *t*. Thus, when the cells leaving the live cell population have a smaller (larger) average lipid load than the live cells that remain, the average lipid load of the live cell population tends to increase (decrease). The impact of apoptosis and emigration on ${{\bar{A}}}_M\left( t\right)$ observed here is entirely due to the lipid-dependent terms in the model. In the absence of lipid-dependent apoptosis and emigration, the final two terms on the right-hand side of (35) equate to zero.

For ${{\bar{A}}}_P\left( t\right)$, we have:

36 $$\begin{aligned} \frac{d{{\bar{A}}}_P\left( t\right) }{dt} = \frac{M\left( t\right) }{P\left( t\right) }\Big [G_{\beta a}\left( t\right) - G_\beta \left( t\right) {{\bar{A}}}_P\left( t\right) \Big ]. \end{aligned}$$

This equation contains only a term relating to macrophage apoptosis. We see that ${{\bar{A}}}_P\left( t\right)$ increases when $\frac{G_{\beta a}\left( t\right) }{G_\beta \left( t\right) } > {{\bar{A}}}_P\left( t\right)$ and decreases when $\frac{G_{\beta a}\left( t\right) }{G_\beta \left( t\right) } < {{\bar{A}}}_P\left( t\right)$. This makes sense because ${{\bar{A}}}_P\left( t\right)$ increases (decreases) when the average lipid load of dying cells is larger (smaller) than the average lipid load of those already dead. Unlike the corresponding term in the ${{\bar{A}}}_M\left( t\right)$ equation, the right-hand side here does not vanish in the absence of lipid-dependent apoptosis. Instead, the term in brackets reduces to ${{\bar{A}}}_M\left( t\right) - {{\bar{A}}}_P\left( t\right)$ and the ODE acts to equilibrate the average apoptotic cell lipid content to the average live cell lipid content.

## Appendix C: Numerical Solution Methods

The model equations are solved by reformulating (23)–(29) as a large system of coupled ODEs (method of lines) and integrating with the MATLAB routine *ode15s*. This approach requires the semi-infinite *a* domain for Eqs. (23) and (24) to be truncated at a finite upper limit $a_{max}$ and appropriately discretised. In choosing $a_{max}$, we aim to minimise numerical error associated with the loss of live cells (and their ingested lipid) across the upper domain boundary. For the scenarios considered in this paper, this typically requires $a_{max}$ values on the order $10^2$–$10^3$. Since uniform discretisation of such large domains would require a substantial (and potentially unfeasible) number of grid points, we adopt a non-uniform gridding strategy to reduce the number of points required.

We discretise the *a* domain into *I* points $a_i$ ($i = 1,2,\ldots ,I$) by assuming that the spacing between adjacent points $\Delta a_i = a_{i+1} - a_i$ increases linearly from $\Delta a_1 = \Delta a_{min}$ to $\Delta a_{I-1} = c\Delta a_{min}$, where $c > 1$ is a constant. (This equates to adding the constant increment $\frac{\Delta a_{min} \left( c-1\right) }{\left( I-2\right) }$ to the width of each subsequent grid spacing). The positions of individual grid points are thus given by the expression:

37 $$\begin{aligned} a_i = 1 + \Delta a_{min} \left( i-1\right) \left[ 1 + \frac{\left( i-2\right) \left( c-1\right) }{2\left( I-2\right) }\right] , \end{aligned}$$

where $a_{max} = a_I = 1 + \frac{1}{2} \Delta a_{min} \left( c + 1\right) \left( I - 1\right)$. This non-uniform gridding approach is by no means optimised for the problem. However, we find that it is well suited to the model equations because it allows for the use of a high grid point density near $a = 1$ (where solutions can vary rapidly with *a*) and a low grid point density for $a \gg 1$ (where solutions have relatively little variation with *a*). For the simulations in this paper, we use two different domain sizes with the following discretisations:

1. $$\Delta a_{min} = 0.005$$, $$c = 126$$, $$I = 1258$$ $$\implies a_{max} = 400.0975$$;
2. $$\Delta a_{min} = 0.005$$, $$c = 200$$, $$I = 2001$$ $$\implies a_{max} = 1006$$.

Note that the ratios $\frac{\Delta a_{min} \left( c-1\right) }{\left( I-2\right) }$ are very similar for the two discretisations, meaning that corresponding points $a_i$ have almost identical positions for all $i \leqslant 1258$. Most simulations in this study use the smaller domain. However, for cases that consider lipid-dependent emigration, calculations are performed on the larger domain. The larger domain is required in this case because the reduced rate of emigration for lipid-loaded cells tends to drive an increase in the proportion of cells with very large lipid quantities.

Using the above discretisation, we define the quantities $m_i\left( t\right) = m\left( a_i,t\right)$ and $p_i\left( t\right) = p\left( a_i,t\right)$ for $i = 1,\ldots ,I$. Equations (23)–(29) are then recast as a system of $2I + 4$ ODEs for $m_2\left( t\right) ,\ldots ,$ $m_I\left( t\right)$, $p_1\left( t\right) ,\ldots ,$ $p_I\left( t\right)$, $N\left( t\right)$, $M\left( t\right)$, $A_M\left( t\right)$, $P\left( t\right)$ and $A_P\left( t\right)$ coupled to an algebraic constraint for $m_1\left( t\right)$ as given by the boundary condition (30). All integral terms in these ODEs are approximated in terms of the $m_i\left( t\right)$ and $p_i\left( t\right)$ using the trapezoidal rule. The derivative in the structural variable is approximated by the non-uniform second-order upwind scheme:

38 $$\begin{aligned} \frac{\partial m}{\partial a}\left( a_i,t\right) \approx \frac{3m_i\left( t\right) - 4m_{i-1}\left( t\right) + m_{i-2}\left( t\right) }{3\Delta a_{i-1} - \Delta a_{i-2}}, \end{aligned}$$

for $3 \leqslant i \leqslant I$, and by the non-uniform second-order centered scheme:

39 $$\begin{aligned} \frac{\partial m}{\partial a}\left( a_2,t\right) \approx \frac{m_3\left( t\right) - m_1\left( t\right) }{\Delta a_2 + \Delta a_1} \end{aligned}$$

for $$i=2$$.

The proliferation and efferocytosis terms in Eq. (23) pose additional challenges to the numerical solution of the ODE system. The following provides further details on our handling of these non-local terms:

1. The proliferation source term $4 \rho g_\rho \left( 2a-1\right) m(2a-1,t)$ is not well-defined for $2a-1 > a_{max}$ (i.e. proliferation of cells with lipid loads greater than $a_{max}$ cannot be quantified). We therefore omit this term from all $m_i\left( t\right)$ equations for which $a_i > \frac{1}{2}\left( a_{max}+1\right)$. For the large simulation domains that we use, the numerical error associated with these omissions is negligible (indeed, in the case that $g_\rho \left( a\right)$ tends rapidly to 0, the error is vanishingly small).
2. To evaluate the proliferation and efferocytosis source terms in the $m_i\left( t\right)$ equations, we often need to approximate *m* and *p* values at positions that are not coincident with grid points (e.g. $m\left( 2a-1,t\right)$ in the proliferation source term, $p\left( a-a',t\right)$ in the integrand of the efferocytosis source term). Whenever such an approximation is required, we perform a linear interpolation using the appropriate pair of $m_i\left( t\right)$ or $p_i\left( t\right)$ values from adjacent grid points.
3. Upon each evaluation of an efferocytosis source integral, we divide the result by the numerical approximation to the following integral:
  $$\begin{aligned} \int _{1}^{\infty }\int _{1}^{a-1} m(a',t) p(a-a',t) \, da' \, da = \left( \int _{1}^{\infty } m\left( a,t\right) \, da\right) \cdot \left( \int _{1}^{\infty } p\left( a,t\right) \, da\right) . \end{aligned}$$
  This re-normalises the result and acts to prevent the growth of small errors that arise in the numerical calculation of the efferocytosis integral. No such intervention is required for the other integral terms in the model.
